# Flexible regulation of CRISPR/Cas12a activity by spatial confinement effect

**DOI:** 10.1093/nar/gkag414

**Published:** 2026-05-04

**Authors:** Huan Yang, Bo Shen, Yuwei Wang, Jianxiong Liu, Fangzhu Zhou, Min Liu, Jie Li, Jinjin Fan, Shijia Ding, Jinlin Guo, Juan Zhang, Xinmin Li

**Affiliations:** College of Medical Technology, Chengdu University of Traditional Chinese Medicine, Chengdu 611137, China; Department of Laboratory Medicine, Chongqing Hospital of Traditional Chinese Medicine, Chongqing 400021, China; Department of Laboratory Medicine, Chongqing Hospital of Traditional Chinese Medicine, Chongqing 400021, China; Key Laboratory of Clinical Laboratory Diagnostics (Ministry of Education), College of Laboratory Medicine, Chongqing Medical University, Chongqing 400016, China; Department of Laboratory Medicine, Chongqing Hospital of Traditional Chinese Medicine, Chongqing 400021, China; College of Medical Technology, Chengdu University of Traditional Chinese Medicine, Chengdu 611137, China; Department of Laboratory Medicine, Chongqing Hospital of Traditional Chinese Medicine, Chongqing 400021, China; Key Lab for Special Functional Materials of Ministry of Education, Henan University, Kaifeng 475004, China; Key Laboratory of Clinical Laboratory Diagnostics (Ministry of Education), College of Laboratory Medicine, Chongqing Medical University, Chongqing 400016, China; College of Medical Technology, Chengdu University of Traditional Chinese Medicine, Chengdu 611137, China; College of Medical Technology, Chengdu University of Traditional Chinese Medicine, Chengdu 611137, China; Department of Laboratory Medicine, Chongqing Hospital of Traditional Chinese Medicine, Chongqing 400021, China; Department of Laboratory Medicine, Chongqing Hospital of Traditional Chinese Medicine, Chongqing 400021, China

## Abstract

Precise regulation of the trans-cleavage activity of CRISPR/Cas12a has substantially expanded its utility in molecular diagnostics. However, existing strategies rely predominantly on systems with freely diffusing components, necessitating intricate CRISPR RNA (crRNA) designs or specialized chemical modifications, which hinder their simplicity and broader applicability. Here, we demonstrate that the activity of spatially confined Cas12a on fluid membranes (CAS-FLIER) can be facilely modulated by simply adjusting the length of crRNA and the duplex-strand reporters. We reveal that fine-tuning the movement range of membrane-Cas12a and the accessibility of the reporter to Cas12a enables precise, scalable control over trans-cleavage activity. As a proof of concept, we show that the activity of confined Cas12a can be co-activated by single-stranded DNA (ssDNA) and RNA inputs, a capability that remains unattainable in conventional freely diffusing systems. Furthermore, by incorporating a DNA reverse-transcriptor into the CAS-FLIER system, we achieve one-pot, highly sensitive detection of HIV RNA, supporting accurate diagnosis of HIV infection. Notably, this assay is compatible with a lateral-flow format for direct visual readout, highlighting its potential as a point-of-care diagnostic tool for HIV. Collectively, our findings shed new light on modulating Cas12a activity, advancing its applications in molecular diagnostics.

## Graphical Abstract

**Graphical Abstract:**
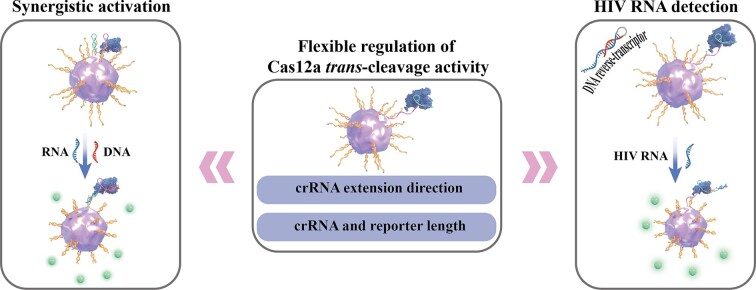

## Introduction

Clustered regularly interspaced short palindromic repeats (CRISPR/Cas12a) systems, characterized by the binding of CRISPR RNA (crRNA)-Cas12a ribonucleoprotein to target nucleic acids, activating indiscriminate trans-cleavage activity for single-stranded DNA (ssDNA) [1, 2, 3], have been widely explored for diagnostic applications [4–14]. However, neither standalone CRISPR/Cas12a nor its direct integration with signal amplification technologies can simultaneously achieve the high sensitivity required for low-abundance targets and the operational simplicity demanded for clinical translation [15–18]. To address this limitation, precise and programmable control over the trans-cleavage activity of CRISPR/Cas12a systems is crucial for enhancing their compatibility and sensitivity, advancing their applications.

Currently, various strategies have been explored to regulate Cas12a function, including crRNA structural engineering, chemical modification of crRNA, and DNA framework switches. For example, modifications to the 3′ end of crRNA repeat region [19, 20], crRNA binding to overhanging activator [21], and circular crRNA design [22] have expanded Cas12a’s sensing scope to small molecules, proteins, and bacteria. Light-activated crRNA [23–28], photocleavable DNA-tethered crRNA [29], DNA/RNA-blocked crRNA [30, 31], G-quadruplex-containing crRNA [32] have improved compatibility with isothermal nucleic acid amplification, thereby boosting detection sensitivity. However, these approaches are plagued by inherent drawbacks. First, elaborate crRNA architectures complicate sequence design. Second, specialized chemical modifications often require ultraviolet radiation or enzyme digestion, which can compromise Cas12a activity. Third, imperfect stoichiometry or annealing during crRNA-complementary strand prehybridization frequently results in inefficient activity modulation. While DNA framework switches mitigate some of these issues, their regulatory capacity remains largely dependent on input concentration [33–36]. Notably, nearly all reported strategies rely on freely diffusing components, which inherently restricts the precision and tunability of Cas12a activity control.

In multicellular organisms, cell membranes act as dynamic, confined interfaces that orchestrate the activity of embedded proteins, enabling the precise coordination of cellular processes and physiological functions [37, 38]. For example, the B cell receptor, a transmembrane protein complex, undergoes synergistic activation by antigens and complement fragments to initiate B cell-mediated immune responses [39]. Beyond their physiological roles, cell membranes enhance molecular collision frequencies in confined spaces, accelerating biochemical reaction kinetics [40–43]. Notably, this spatial confinement effect has been harnessed to boost the trans-cleavage activity of CRISPR/Cas12a [44, 45], prompting the hypothesis that fluidic, confined membrane architectures can serve as a versatile platform for highly scalable regulation of Cas12a activity.

Here, we present CAS-FLIER (CRISPR/Cas12a on fluid membranes for activity regulation), a strategy that exploits spatial confinement to achieve flexible, programmable control of Cas12a trans-cleavage. We show that three parameters, crRNA extension direction and length, and the length of double-stranded reporters with sticky ends, exert a profound influence on Cas12a activity when localized to membranes, but have only marginal effects in freely diffusing systems. Furthermore, we demonstrate that CAS-FLIER can be synergistically activated by single-stranded DNA (ssDNA) and RNA, with RNA specifically modulating the movement range of Cas12a. By integrating a DNA reverse-transcriptor that converts specific RNA hybridization events into ssDNA activators, we enable one-pot, rapid, and highly sensitive detection of HIV RNA, a key advance for the accurate diagnosis of HIV infection. Finally, coupling CAS-FLIER with a lateral flow strip assay (LFA) validates its potential as a robust point-of-care (POC) tool for clinical diagnostics.

## Materials and methods

### Ethics and inclusion statement

Healthy human whole blood, HIV-positive samples (*n* = 12), and healthy control samples (*n* = 8) were obtained from Chongqing Hospital of Traditional Chinese Medicine, in compliance with an approved Institutional Review Board (IRB) protocol (Scientific Ethics Approval No.: 2025-YJS-HY-2). All clinical samples were collected after obtaining written informed consent from all participants. All patient samples included in this study were de-identified, and individual patient demographic information was not available.

### Materials and reagents

HPLC-purified oligonucleotides (Supplementary Table S1), TE buffer, RNase inhibitor, 10 × phosphate-buffered saline (PBS), RNase-free water, and all DNase/RNase-free tips were purchased from Sangon Biotech Co., Ltd. (Shanghai, China). 6 × loading buffer and 20 bp DNA Marker were obtained from Takara Biotech Inc. (Dalian, China). EnGen LbCas12a (100 μM), Cas12a diluent, 10 × NEBuffer 2.1, and 25 mM MgCl₂ solution were purchased from New England Biolabs Inc. (Ipswich, MA, USA). Nucleic acid test strips (Biotin-FAM) for the CRISPR-Cas system were purchased from Shanghai Tooman Biotechnology Co., Ltd. (Shanghai, China). A Nucleic Acid Extraction Kit (Equipment No.: 20 150 021) and a Human Immunodeficiency Virus (HIV-1) Nucleic Acid Quantitative Detection Kit (PCR-fluorescence probe method; Equipment No.: 20 150 036) were acquired from Sansure Biotech Inc. (Changsha, China).

### Apparatus

All fluorescence spectra and kinetic fluorescence analyses were performed using an F-4700 fluorescence spectrophotometer (Hitachi Scientific Instruments Beijing Co., Ltd., Beijing, China). Gel electrophoresis was performed with an electrophoresis system (Bio-Rad Laboratories, Singapore). Gel images were acquired using a Bio-Rad ChemiDoc XRS imaging system (Bio-Rad Laboratories, Hercules, CA, USA). Fluorescence micrographs were captured with a SOPTOP-XD inverted fluorescence microscope (SOPTOP Optical Instrument Co., Ltd., China). Flow cytometry (FCM) analyses were conducted on a CytoFLEX flow cytometer (Beckman Coulter, Fullerton, CA, USA).

### Construction of spatially confined Cas12a on fluid membranes

Red blood cell (RBC) suspensions were prepared as follows: 200 μL of human whole blood with an initial RBC concentration of 4.19 × 1012 cells/L was obtained from Chongqing Hospital of Traditional Chinese Medicine, and centrifuged at 3500 rpm for 5 min at 4°C, followed by careful removal of the supernatant plasma. The resulting packed RBCs were washed thoroughly four times with 0.01 M phosphate-buffered saline (PBS, pH 7.4). A 5 μL aliquot of the washed, packed RBCs was resuspended in 1000 μL of the same PBS buffer to prepare a 0.5% (v/v) RBC suspension, with a final calibrated concentration of 5.25 × 1010 cells/L.

All oligonucleotide strands were dissolved in TE buffer and stored at a stock concentration of 100 μM. For the preparation of double-stranded reporter probes, 50 μL of cholesterol- and FAM-labelled single-stranded oligonucleotides (10 μM) were mixed with 50 μL of cholesterol- and BHQ1-labelled complementary single strands (10 μM). The mixture was heated to 95°C for 5 min and then slowly cooled to room temperature to allow annealing into double-stranded reporters. All hairpin-structured nucleic acid strands were subjected to the same thermal denaturation protocol (95°C for 5 min, followed by gradual cooling to room temperature) to facilitate the formation of correctly folded hairpin conformations.

The CAS-FLIER system was assembled by mixing the following components in a single reaction mixture: 5 μL of the prepared RBC suspension, 5 μL of double-stranded reporter probes (at various concentrations), 2 μL of cholesterol-modified crRNA (at various concentrations), 20 μL of 10 × NEBuffer 2.1, 2 μL of Cas12a nuclease, and 66 μL of DEPC-treated water. The concentrations of Cas12a and cholesterol-modified crRNA were kept equimolar in each reaction mixture. After incubation at room temperature for 15 min, the mixture was centrifuged at 3500 rpm for 5 min to remove unbound excess nucleic acids and proteins. The resulting CAS-FLIER system was gently resuspended in 100 μL of 2 × NEBuffer 2.1 and stored at 4°C for subsequent experimental use.

### Quantification of reporter molecules anchored on individual RBCs

To determine the number of reporter molecules anchored on each RBC, a standard calibration curve was established by quantifying the fluorescence intensity of cholesterol- and FAM-dual-labeled reporters at serial concentrations (Supplementary Fig. S1). In a typical binding assay, 200 nM of cholesterol- and FAM-dual-labeled reporter was incubated with 5 μL of the prepared RBC suspension (5.25 × 1010 cells/L) for 15 min. Following centrifugation, the fluorescence intensity of the supernatant was measured to be 39.88. Using the linear regression equation Y = 1.30X − 2.90 (where Y denotes fluorescence intensity and X denotes reporter concentration in nM), the concentration of unbound reporter in the supernatant was calculated to be 32.91 nM. Based on this value, the loading efficiency of the reporter onto RBCs was determined to be 83.55%. Assuming a homogeneous distribution of nucleic acid molecules in the reaction system and equivalent accessibility of the probes to RBC membranes, cholesterol-modified oligonucleotide strands bound to RBC membranes in a manner analogous to cholesterol- and FAM-dual-labeled reporter strands. Given that the calibrated number of RBCs was 2.625 × 105 cells, the average number of reporter molecules anchored per RBC was estimated by normalizing the total bound reporter molecules to the total number of RBCs in the reaction system. This calculation revealed that an average of ∼1.92 × 10⁶ reporter molecules was attached to each RBC.

### The FCM and confocal fluorescence imaging analysis

To verify the binding of cholesterol-labeled nucleic acid strands to RBC membranes, control and experimental samples were prepared as follows. The control sample contained 5 μL of FAM-labeled 28-nt reporter (1 μM) and 5 μL of FAM-labeled 5′-poly-U20-crRNA (1 μM). The experimental sample contained 5 μL of FAM- and cholesterol-dual-labeled 28-nt reporter (1 μM) and 5 μL of FAM- and cholesterol-dual-labeled crRNA (1 μM). Each sample was individually mixed with 5 μL of prepared RBC suspension in 2 × NEBuffer 2.1 to a final volume of 100 μL, followed by incubation at room temperature for 15 min. All samples were then washed three times with 0.01 M PBS via centrifugation at 3500 rpm. After the final wash, samples were resuspended in 2 × NEBuffer 2.1 for subsequent FCM analysis and confocal fluorescence imaging.

To validate the operational feasibility of the CAS-FLIER system, 1 μL of target DNA (10 nM) was incubated with 98 μL of the preassembled CAS-FLIER system (containing 4 nM membrane-anchored 5′-poly-U20-crRNA, 4 nM membrane-localized Cas12a, and 200 nM membrane-anchored 38-nt reporters) and 1 μL of 2 × NEBuffer 2.1 at 37°C for 30 min. The untreated CAS-FLIER system served as the control group. Subsequently, these samples were analyzed via FCM and confocal fluorescence imaging.

### Michaelis − Menten enzyme kinetic measurements

Cas12a enzymatic kinetics within the CAS-FLIER, reporter-localized CRISPR/Cas12a, and dispersed CRISPR/Cas12a system were evaluated under conditions of fixed concentrations of Cas12a (10 nM), crRNA (10 nM), and target ssDNA (10 nM), alongside serial concentrations of membrane-immobilized reporter substrate. Reporter constructs of distinct lengths (28, 33, 38, and 43 nt) were applied, with their surface-anchored concentrations on RBC membranes ranging from 10, 30, 60, 120, 240, and 500 nM to 1 μM. All enzymatic reactions of the CAS-FLIER and reporter-localized CRISPR/Cas12a system were carried out in 2 × NEBuffer 2.1. The enzymatic reactions of the dispersed CRISPR/Cas12a system were carried out in 1 × NEBuffer 2.1. Membrane-immobilized reporter concentrations were quantified via fluorescence intensity readings of cholesterol- and FAM-dual-tagged reporter probes pre- and post-incubation with RBCs, with values extrapolated from the matched fluorescence calibration curve. Concentrations of membrane-anchored crRNA were similarly determined using a calibration curve of cholesterol- and Cy5-dual-labeled crRNA. All reactions were performed in technical triplicate at 37 °C on an F-4700 fluorescence spectrophotometer, with fluorescence acquisitions at 30 s intervals. Background-subtracted fluorescence intensities were generated by deducting time-averaged signals of buffer-only control samples from raw experimental fluorescence readouts. Initial reaction velocities (arbitrary units per second, AU s⁻¹) were extracted via linear regression fitting of fluorescence data collected over the first 600 s for each reporter concentration gradient. A calibration curve of cholesterol- and FAM-dual-tagged reporter was further utilized to convert velocity units from AU s⁻¹ to nM s⁻¹. Plots of measured reaction velocities against membrane-immobilized substrate concentrations were fitted to the Michaelis − Menten kinetic model (Eq 4) using GraphPad Prism software (GraphPad Software, CA, USA), enabling derivation of the catalytic constant (kcat) and Michaelis constant (KM).


(1) \begin{eqnarray*}
{\mathrm{E + S}} \rightleftharpoons {\mathrm{E}}{{{\mathrm{S}}}^{{{{\mathrm{k}}}_{{\mathrm{cat}}}}}} \to {\mathrm{E + P}}
\end{eqnarray*}

(2) \begin{eqnarray*}
V = \frac{{{{V}_{{\mathrm{mas}}}}\left[ S \right]}}{{{{K}_M} + \left[ S \right]}}
\end{eqnarray*}

(3) \begin{eqnarray*}
{{k}_{{\mathrm{cat}}}} = \frac{{{{V}_{\max }}}}{{{{{\left[ {\mathrm{E}} \right]}}_0}}}
\end{eqnarray*}

(4) \begin{eqnarray*}
\frac{{{\mathrm{d}}\left[ {\mathrm{P}} \right]}}{{{\mathrm{d}}t}} \approx \frac{{{{k}_{{\mathrm{cat}}}}}}{{{{K}_{\mathrm{M}}}}}\left( {{{{\mathrm{S}}}_0} - \left[ P \right]} \right)
\end{eqnarray*}

n this trans-cleavage reaction, target-activated crRNA–Cas12a complexes (E) mediate the cleavage of intact membrane-bound reporter substrates (S) into fluorescent products (P), with product accumulation monitored via fluorescence quantification. Key enzymatic kinetic parameters, including the catalytic rate constant (kcat), Michaelis constant (KM), and catalytic efficiency (kcat/KM), were then computed from the Michaelis–Menten fitting results.

### Fluorescence spectroscopy quantitative assays

For the feasibility analysis of CAS-FLIER, 1 μL of target DNA (10 nM) was mixed with 98 μL of the preassembled CAS-FLIER (containing 4 nM membrane-anchored 5′-poly-U20-crRNA, 4 nM membrane-localized Cas12a, and 200 nM membrane-anchored 38-nt reporters) and 1 μL of 2 × NEBuffer 2.1. The reaction mixture was incubated at 37°C for 30 min.

For the buffer optimization assay, the preassembled CAS-FLIER (comprising 4 nM membrane-anchored 5′-poly-U20-crRNA, 4 nM membrane-localized Cas12a, and 200 nM membrane-anchored 38-nt reporters) was used, with other reaction components consistent with the above detection protocol except for the tested buffer types.

For the reporter-to-crRNA ratio optimization assay, the concentration of membrane-anchored 5′-poly-U20-crRNA was fixed at 4 nM, while the concentration of membrane-anchored 38-nt reporters was varied to establish different reporter-to-crRNA molar ratios. Other reaction conditions were identical to those of the standard target DNA detection protocol described above.

For the detection of synthetic HIV RNA or HIV RNA analogues using the CAS-FLIER system, reactions were performed as follows. First, 1 μL of DNA reverse-transcriptor (10 μM) was mixed with 98 μL of the preassembled CAS-FLIER system (containing 4 nM membrane-anchored 5′-poly-U20-crRNA, 4 nM membrane-localized Cas12a, and 200 nM membrane-anchored 38-nt reporters). Subsequently, 1 μL of synthetic HIV RNA (at gradient concentrations) or HIV RNA analogues (10 nM) was added to the mixture. Following incubation at 37°C for 30 min, the fluorescence intensity of each reaction mixture was measured.

For synthetic HIV RNA detection using the reporter-localized CRISPR/Cas12a system, reactions were conducted in 2 × NEBuffer 2.1, with concentrations of Cas12a, 5′-poly-U20-crRNA, and membrane-bound 38-nt reporters set to 4, 4, and 200 nM, respectively. For detection using the dispersed CRISPR/Cas12a system, reactions were carried out in 1 × NEBuffer 2.1, with Cas12a, crRNA, and 38-nt reporters maintained at 4, 4, and 200 nM, respectively. All other detection procedures were identical to those described for the CAS-FLIER system.

All fluorescence spectra were acquired using an F-4700 fluorescence spectrophotometer, with excitation at 490 nm and emission scanning over the range of 500–600 nm. Quantitative analysis of target nucleic acids was performed by quantifying the fluorescence intensity at the emission maximum of 520 nm.

### Real-time fluorescence testing of CAS-FLIER (double-target responsive system) and freely dispersed CRISPR/Cas12a system

For the CAS-FLIER system, 1 μL of synthetic HIV RNA (1 nM) and/or 1 μL of synthetic ssDNA (1 nM) was mixed with 98 μL of the preassembled CAS-FLIER system (containing 4 nM membrane-anchored hairpin-structured crRNA, 4 nM membrane-localized Cas12a, and 200 nM membrane-anchored 38-nt reporters). Each reaction mixture was adjusted to a final volume of 100 μL with 2 × NEBuffer 2.1 if necessary.

For the freely dispersed CRISPR/Cas12a system, 1 μL of synthetic HIV RNA (1 nM) and/or 1 μL of synthetic ssDNA (1 nM) was mixed with 98 μL of the Cas12a system prepared in 1 × NEBuffer 2.1 (containing 4 nM hairpin-structured crRNA, 4 nM Cas12a, and 200 nM 38-nt reporters). Reactions were brought to a final volume of 100 μL with 1 × NEBuffer 2.1 as required.

Immediately after sample preparation, real-time fluorescence intensities were measured using an F-4700 fluorescence spectrophotometer with excitation at 490 nm and emission at 520 nm. Fluorescence signals were collected at 1 s intervals.

Native polyacrylamide gel electrophoresis (PAGE)

To analyze the hybridization between the DNA reverse-transcriptor (H1) and synthetic HIV RNA, H1 and synthetic HIV RNA were mixed at an equimolar concentration of 1 μM and incubated at 37°C for 30 min. For investigating Cas12a-mediated reporter cleavage, all substrates were used at 1 μM, and the cleavage reaction was conducted at 37°C for 30 min.

A 12% native polyacrylamide gel was prepared in 1 × TBE buffer (89 mM Tris, 89 mM boric acid, 2 mM EDTA, pH 8.3). Aliquots of 10 μL per sample (containing different substrates) were loaded onto the gel wells. Electrophoresis was performed at 110 V at room temperature for 55 min using an electrophoresis system. Following staining with gel dye for 20 min, gels were imaged using a Bio-Rad ChemiDoc XRS imaging system (Bio-Rad Laboratories, Hercules, CA, USA).

### Clinical sample assays

Clinical blood samples were first centrifuged at 3500 g for 15 min to isolate serum. Total RNA was then extracted from the collected serum samples following the manufacturer’s instructions of the Nucleic Acid Extraction Kit. The extracted RNA was subjected to detection using the CAS-FLIER system (containing 4 nM membrane-anchored 5′-poly-U20-crRNA, 4 nM membrane-localized Cas12a, and 200 nM membrane-anchored 38-nt reporters) and quantitative reverse transcription-polymerase chain reaction (qRT-PCR). The detection procedure for extracted RNA using the CAS-FLIER system was identical to that described for synthetic HIV RNA. qRT-PCR was performed in accordance with the manufacturer’s protocol of the Human Immunodeficiency Virus (HIV-1) Nucleic Acid Quantitative Detection Kit.

For the lateral flow strip assay, 1 μL of extracted RNA was added to a 49 μL preassembled reaction system containing the CAS-FLIER components (4.8 nM membrane-anchored 5′-poly-U20-crRNA, 4.8 nM membrane-localized Cas12a, 240 nM membrane-anchored 38-nt reporters) and DNA reverse-transcriptor (100 nM). The mixture was incubated at 37°C for 30 min, after which 50 μL of the reaction mixture was applied to the lateral flow strip. Results were visualized and recorded after 5 min of incubation.

### Statistical analysis

All data are presented as mean ± standard deviation (s.d.) from three independent experiments. The mean and standard deviation were calculated using standard formulas. Differences between the two groups were evaluated using a two-tailed unpaired Student’s t-test. Data analysis was performed using GraphPad Prism software.

## Results and discussion

### CAS-FLIER: localized CRISPR/Cas12 system on RBC membrane

**Scheme 1.**
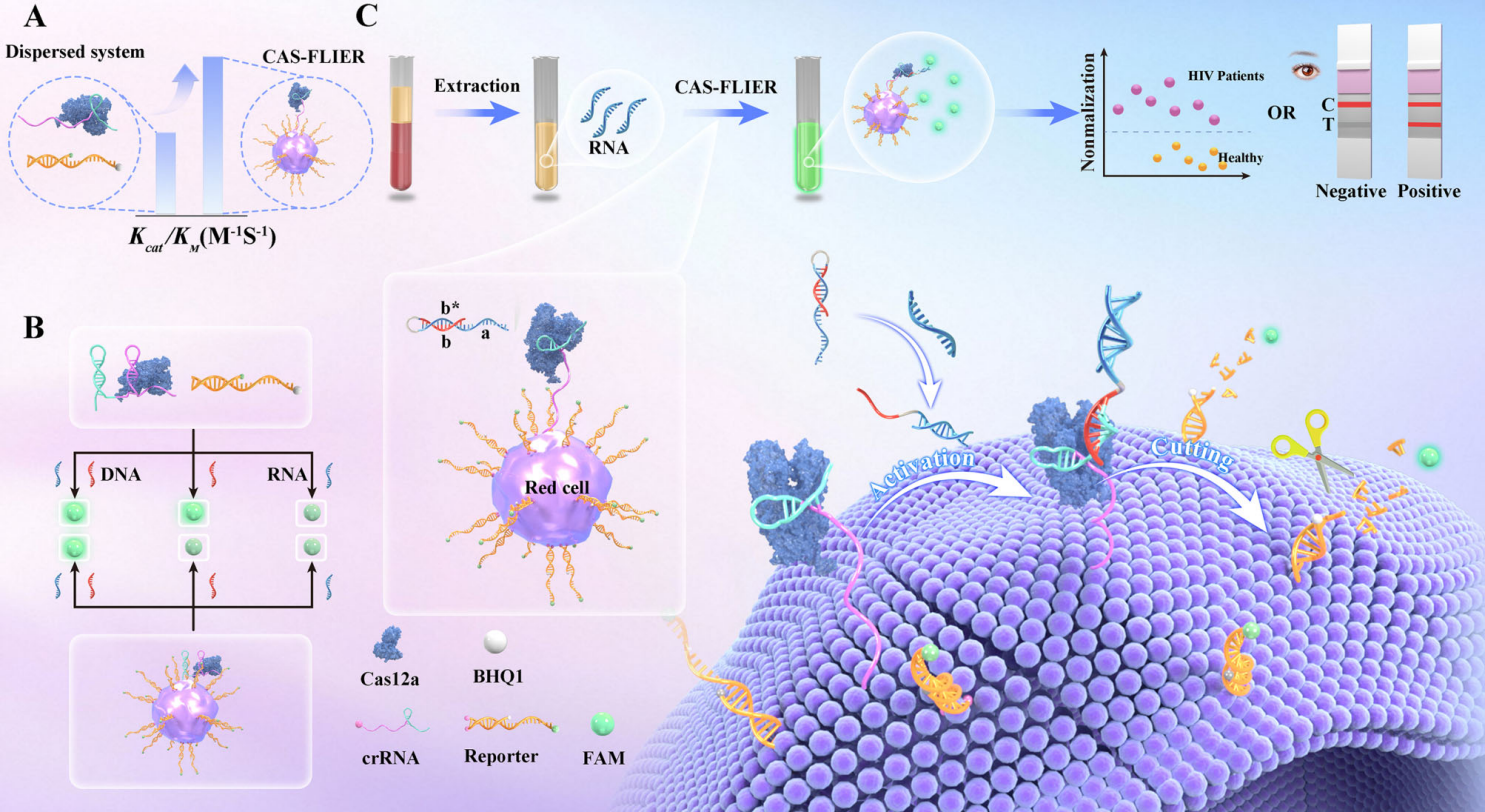
Schematic illustration of CAS-FLIER. (**A**) Compared with freely dispersed CRISPR/Cas12a systems, CAS-FLIER enables highly scalable, programmable regulation of Cas12a trans-cleavage activity. (**B**) CAS-FLIER achieves synergistic activation dependent on both RNA and single-stranded DNA (ssDNA) via a hairpin-containing crRNA that restricts Cas12a movement on the membrane. Concurrent presence of dual targets unwinds the crRNA hairpin and activates Cas12a. (**C**) CAS-FLIER enables one-pot, highly sensitive detection of HIV RNA and compatibility with lateral flow assay (LFA) for point-of-care (POC) diagnostics through a DNA reverse-transcriptor with hairpin structure. A toehold-mediated strand displacement reaction between the probe and HIV RNA induces a hairpin-to-duplex structural transition, activating membrane-anchored Cas12a. Activated Cas12a mediates continuous cleavage of adjacent membrane-immobilized reporters, resulting in fluorescence signal release.

To enable highly scalable regulation of Cas12a activity, we developed CAS-FLIER, a CRISPR/Cas12a system comprising Cas12a protein, crRNA, and reporter strands, which is immobilized on the surface of RBC membranes via hydrophobic interactions between cholesterol moieties and the lipid bilayer (Scheme 1). We designed a double-stranded reporter with sticky ends that fulfills two critical functions: enhancing structural rigidity and providing Cas12a-cleavable sticky termini. Specifically, one strand is conjugated to 6-carboxyfluorescein (FAM) and cholesterol, while the complementary strand is modified with Black Hole Quencher-1 (BHQ1) and cholesterol. In the presence of nucleic acid activators, membrane-immobilized Cas12a mediates the continuous cleavage of adjacent reporters, thereby restoring the fluorescence signal of FAM.

Compared with freely dispersed CRISPR/Cas12a systems, CAS-FLIER offers distinct advantages for modulating Cas12a activity, enabling synergistic activation by RNA and ssDNA, and facilitating highly sensitive RNA detection.

First, CAS-FLIER confers programmable control over Cas12a trans-cleavage. Within this localized membrane environment, Cas12a activity can be tuned through three key parameters: [1] adjusting crRNA length to modulate the movement range of Cas12a on the membrane, [2] altering the crRNA extension direction to reorient the RuvC domain of Cas12a, and [3] varying the reporter length to regulate the accessibility of Cas12a to the reporter substrate. Notably, these factors exert only marginal effects in freely dispersed systems. Therefore, CAS-FLIER achieves highly scalable, programmable regulation of Cas12a activity without elaborate crRNA design or specialized chemical modifications, overcoming key limitations of conventional freely dispersed CRISPR/Cas12a systems (Scheme 1A).

Second, CAS-FLIER can be configured as a double-target responsive system, requiring the synergistic action of RNA and ssDNA for activation. As shown in Scheme 1B, we designed a crRNA with a hairpin structure to restrict Cas12a movement to a narrow spatial range. Only in the concurrent presence of target RNA and ssDNA does the RNA unwind the crRNA hairpin, endowing Cas12a with the capacity for long-range movement on the membrane. Subsequent binding of ssDNA to the crRNA–Cas12a complex triggers trans-cleavage, leading to fluorescence recovery. In contrast, Cas12a remains inactive when only ssDNA or RNA is present. This enables CAS-FLIER to directly and simultaneously detect two distinct target nucleic acids in a single readout. Notably, this dual-input gating mechanism is unachievable in freely dispersed CRISPR/Cas12a systems, where Cas12a is activated by ssDNA alone.

Third, CAS-FLIER enables one-pot, highly sensitive detection of HIV RNA and is compatible with LFA for POC diagnostics. As illustrated in Scheme 1C, we designed a DNA reverse-transcriptor with hairpin structure comprising a, b, and b* domains, which converts HIV RNA hybridization events into ssDNA activators. The “b” domain is sequestered within the hairpin stem, rendering the probe inactive for triggering Cas12a cleavage. In the presence of target HIV RNA, the RNA first binds to the “a” toehold domain and subsequently unwinds the hairpin probe via a strand displacement reaction. This hairpin-to-duplex structural transition exposes the “b” domain, which then binds to the crRNA–Cas12a complex to activate Cas12a trans-cleavage. The activated Cas12a then mediates rapid, continuous cleavage of the membrane-immobilized reporters. Using RNA extracted from clinical samples, CAS-FLIER achieves highly sensitive quantification of HIV RNA via fluorescence readout and visual detection through LFA integration.

### CAS-FLIER characterization

**Figure 1.**
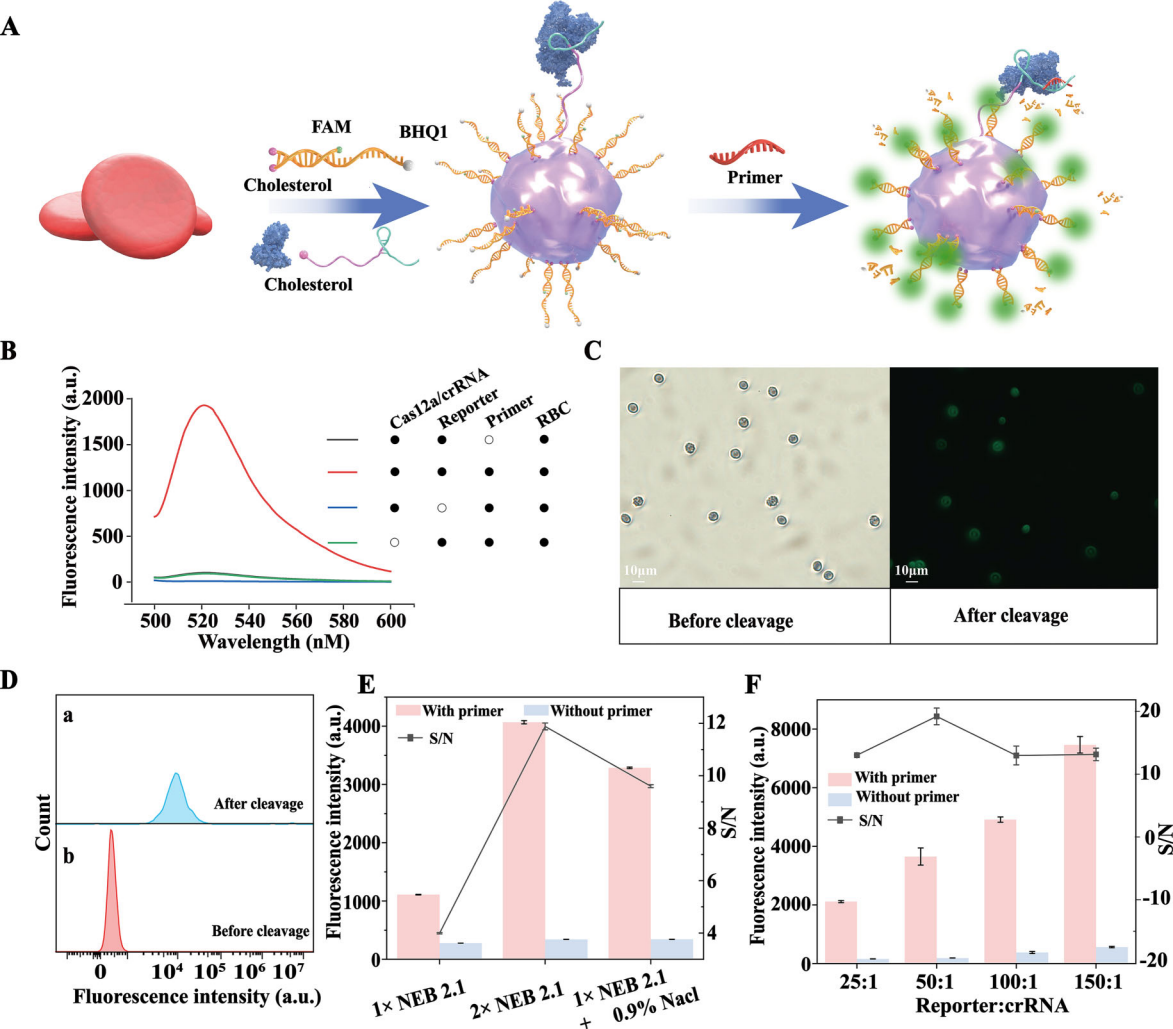
Characterization of CAS-FLIER. (**A**) Schematic illustration of the binding of cholesterol-modified crRNA and double-stranded reporters to red blood cell (RBC) membranes, and subsequent Cas12a activation. (**B**) Representative fluorescence spectra of CAS-FLIER under different experimental conditions. (**C, D**) Representative confocal fluorescence images (**C**) and flow cytometry (FCM) analysis (**D**) of RBCs before and after cleavage of membrane-bound reporters. (**E**) Fluorescence signals of CAS-FLIER in the three tested buffers. (**F**) Effect of the reporter-to-crRNA ratio on CAS-FLIER performance. All experiments were performed with 10 nM target DNA and a 30-min reaction time. Error bars represent mean ± standard deviation (s.d.) from three independent experiments.

We systematically characterized CAS-FLIER. Fig. 1A illustrates the binding of cholesterol-modified crRNA and double-stranded reporters to RBC membranes, as well as subsequent Cas12a activation. Flow cytometry (FCM) and confocal fluorescence imaging confirmed the successful insertion of cholesterol-modified nucleic acid strands into RBC membranes, with distinct fluorescence signals detected in RBCs treated with FAM- and cholesterol-dual-modified crRNA and reporters relative to control groups (RBCs treated with FAM-modified strands alone or untreated RBCs, Supplementary Fig. S2). Subsequently, we observed a strong fluorescent signal from CAS-FLIER in the presence of target DNA (primer), whereas negligible signals were detected in the absence of either reporters or primer (Fig. 1B). Confocal fluorescence imaging and FCM analysis confirmed prominent fluorescence in RBCs within the CAS-FLIER following cleavage of membrane-bound reporters (Fig. 1C and D). Collectively, these results demonstrate the operational feasibility of CAS-FLIER.

During the experimental process, we observed that 1 × NEBuffer 2.1 induced RBC hemolysis (Supplementary Fig. S3). This effect arose because the osmolarity of 1 × NEBuffer 2.1 is lower than that of RBCs, which interferes with CAS-FLIER performance. To address this, we adjusted the osmolarity of the reaction buffer using two formulations: 2 × NEBuffer 2.1 and a buffer containing 1 × NEBuffer 2.1 plus 0.9% NaCl. Notably, CAS-FLIER exhibited no observable hemolysis in either buffer and remained stable at 37°C for 2 h (Supplementary Fig. S3). Furthermore, comparison of the signal-to-noise (S/N) ratios of CAS-FLIER across the three buffers revealed that the system performed optimally in 2 × NEBuffer 2.1. Specifically, the S/N ratio was 2.1-fold higher than that in 1 × NEBuffer 2.1 and 1.2-fold higher than that in 1 × NEBuffer 2.1 plus 0.9% NaCl, respectively (Fig. 1E). Thus, we selected 2 × NEBuffer 2.1 as the final reaction buffer for the CAS-FLIER. Next, we optimized the ratio of reporter to crRNA. As shown in Fig. 1F, as the ratio increased gradually from 25:1 to 150:1, the fluorescence signal exhibited an increasing trend, accompanied by a concurrent gradual elevation of the background signal. The optimal S/N ratio was achieved at a reporter-to-crRNA ratio of 50:1.

### CAS-FLIER achieves highly scalable regulation of Cas12a trans-cleavage activity

**Figure 2.**
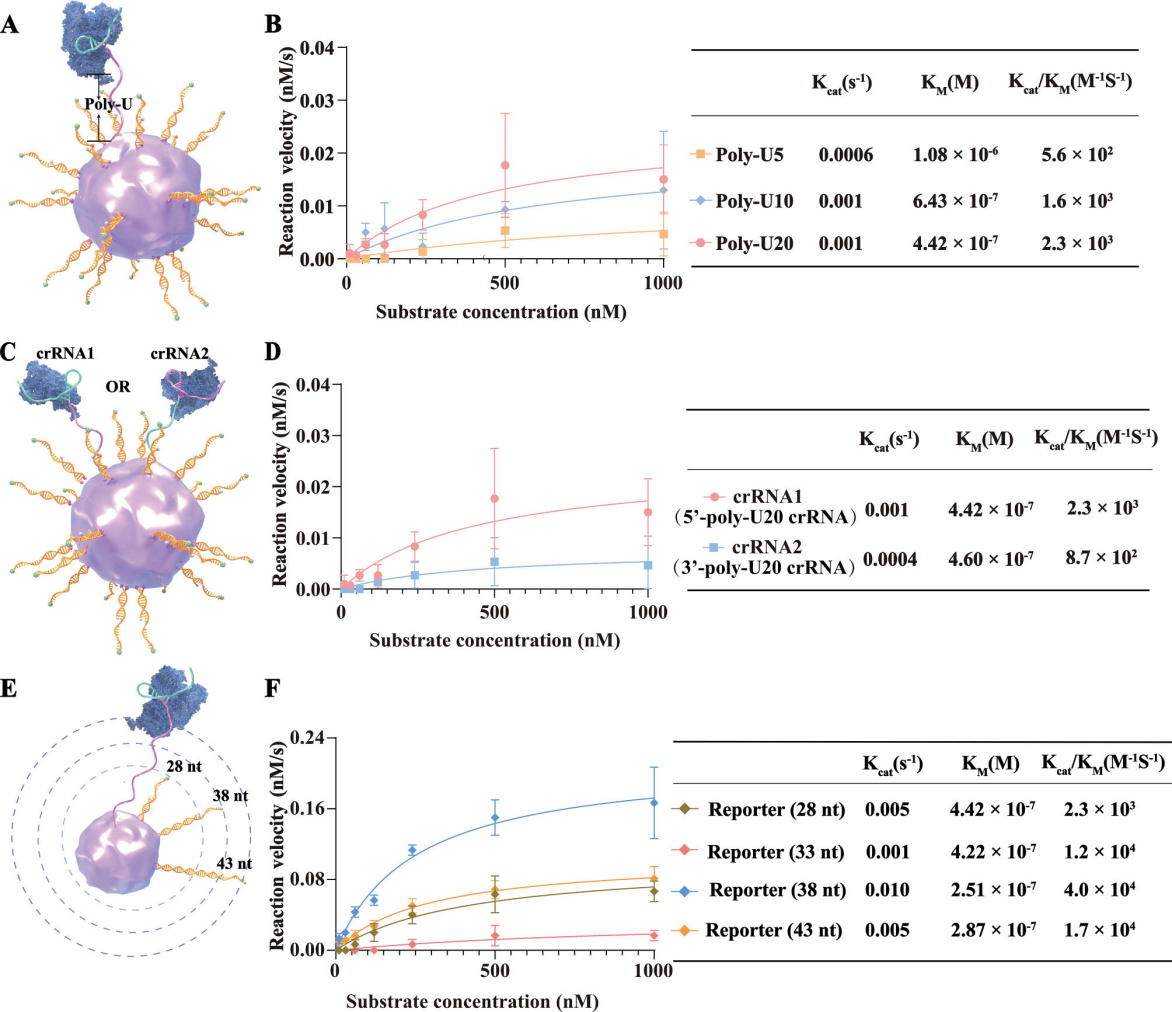
CAS-FLIER-mediated regulation of Cas12a trans-cleavage activity. (**A**) Schematic illustration of crRNA length adjustment via 5′-end extension with polyuracil (poly-U) bases. (**B**) Reaction velocity versus substrate concentration curves (left) and quantitative analysis (right) of Cas12a trans-cleavage activity with 5′-poly-U5, 5′-poly-U10, or 5′-poly-U20 crRNA (20, 10, or 5 consecutive uracils at the crRNA 5′-end, respectively). Poly-U extension enhances Cas12a trans-cleavage activity. Reporter length: 28 nt. (**C**) Schematic illustration of crRNA extension direction (5′- vs 3′-end) on RBC membrane. (**D**) Reaction velocity versus substrate concentration curves (left) and quantitative analysis (right) of Cas12a trans-cleavage activity with 5′-poly-U20 or 3′-poly-U20 crRNA (20 consecutive uracils at the crRNA 5′- or 3′-end, respectively). Cas12a trans-cleavage activity is higher with 5′-poly-U20 crRNA than with 3′-poly-U20 crRNA. Reporter length: 28 nt. (**E**) Schematic illustration of reporters with different lengths anchored on RBC membranes. (**F**) Reaction velocity versus substrate concentration curves (left) and quantitative analysis (right) of Cas12a trans-cleavage activity with 28-, 33-, 38-, or 43-nt reporters (5′-poly-U20 crRNA used). Maximum Cas12a trans-cleavage activity was observed with the 38-nt reporter. All experiments were performed with 10 nM target DNA. Error bars represent mean ± standard deviation (s.d.) from three independent experiments.

Precise modulation of Cas12a activity is critical for expanding its applications [46, 47]. However, existing strategies operate in freely dispersed systems, which suffer from complexity and poor controllability over Cas12a activity [48, 49]. To address this challenge, we developed CAS-FLIER, a platform that enables flexible, straightforward enhancement and suppression of Cas12a trans-cleavage activity. By anchoring Cas12a to the surface of RBC membranes, the movement range of Cas12a is dictated by the length of the crRNA. We thus adjusted crRNA length by extending its 5′-end with polyuracil (Poly-U) bases to investigate the impact on Cas12a trans-cleavage activity (Fig. 2A). As anticipated, extending the crRNA with Poly-U from 5 to 20 nt resulted in a gradual increase in Cas12a trans-cleavage activity (Fig. 2B and Supplementary Fig. S4). Specifically, when using the 5′-Poly-U20 crRNA (20 consecutive uracils), Cas12a exhibited a KM of 4.42 × 10^−7^ M and a kcat/KM of 2.3 × 103 M^−1^ s^−1^, representing a 2.4-fold reduction in KM and a 4.1-fold increase in catalytic efficiency compared to the 5′-poly-U5 crRNA (KM = 1.08 × 10^−6^ M; kcat/KM = 5.6 × 102 M^−1^ s^−1^). The catalytic efficiency of Cas12a with intermediate Poly-U lengths (e.g. poly-U10 or poly-U15) fell between these two lengths (kcat/KM = 1.6 × 103 M^−1^ s^−1^). However, further increasing the Poly-U length beyond 20 nt did not enhance Cas12a trans-cleavage activity (Supplementary Fig. S5), as 5′-Poly-U20 already provides sufficient movement range for membrane-anchored Cas12a. These results confirm that adjusting crRNA length within an appropriate range enables precise control of Cas12a activity.

Next, we explored whether the direction of crRNA extension affects Cas12a activity by extending the crRNA with a poly-U20 at its 3′-end (crRNA2; Fig. 2C). Compared to crRNA1 (5′-poly-U20), crRNA2 reduced Cas12a catalytic efficiency by 2.6-fold (kcat/KM = 8.7 × 102 M^−1^ s^−1^, Fig. 2D and Supplementary Fig. S6). This reduction likely arises from a change in the orientation of Cas12a’s RuvC domain relative to the membrane interface, impairing reporter cleavage. We then investigated the effect of reporter length on Cas12a activity using reporters of 28, 33, 38, and 43 nt (Fig. 2E). Notably, Cas12a catalytic efficiency increased from 2.3 × 103 to 4.0 × 104 M^−1^ s^−1^ as reporter length increased from 28 to 38 nt, but decreased when length exceeded 38 nt (Fig. 2F and Supplementary Fig. S7). Short reporters may be prone to adsorption onto the membrane surface (limiting accessibility to Cas12a), while excessively long reporters may sterically hinder Cas12a movement, contributing to reduced activity. Collectively, CAS-FLIER enables programmable modulation of Cas12a trans-cleavage activity over a ∼70-fold range (from 5.6 × 102 to 4.0 × 104 M^−1^ s^−1^). In principle, combining adjustments to crRNA length, crRNA extension direction, and reporter length allows for highly precise, scalable control of Cas12a activity. Importantly, these parameters have minimal impact on Cas12a trans-cleavage activity in freely dispersed systems, as confirmed by fluorescence kinetic profiling showing only modest signal variations across different conditions (Supplementary Fig. S8).

### CAS-FLIER serves as a double-target responsive system

Given that Cas12a trans-cleavage activity within CAS-FLIER correlates closely with its membrane-associated movement range, we hypothesized that CAS-FLIER could be engineered for synergistic activation by two distinct targets. Specifically, one target would release Cas12a from its membrane-restricted state, while the second would bind to the crRNA spacer (target recognition domain) to trigger Cas12a activity.

**Figure 3.**
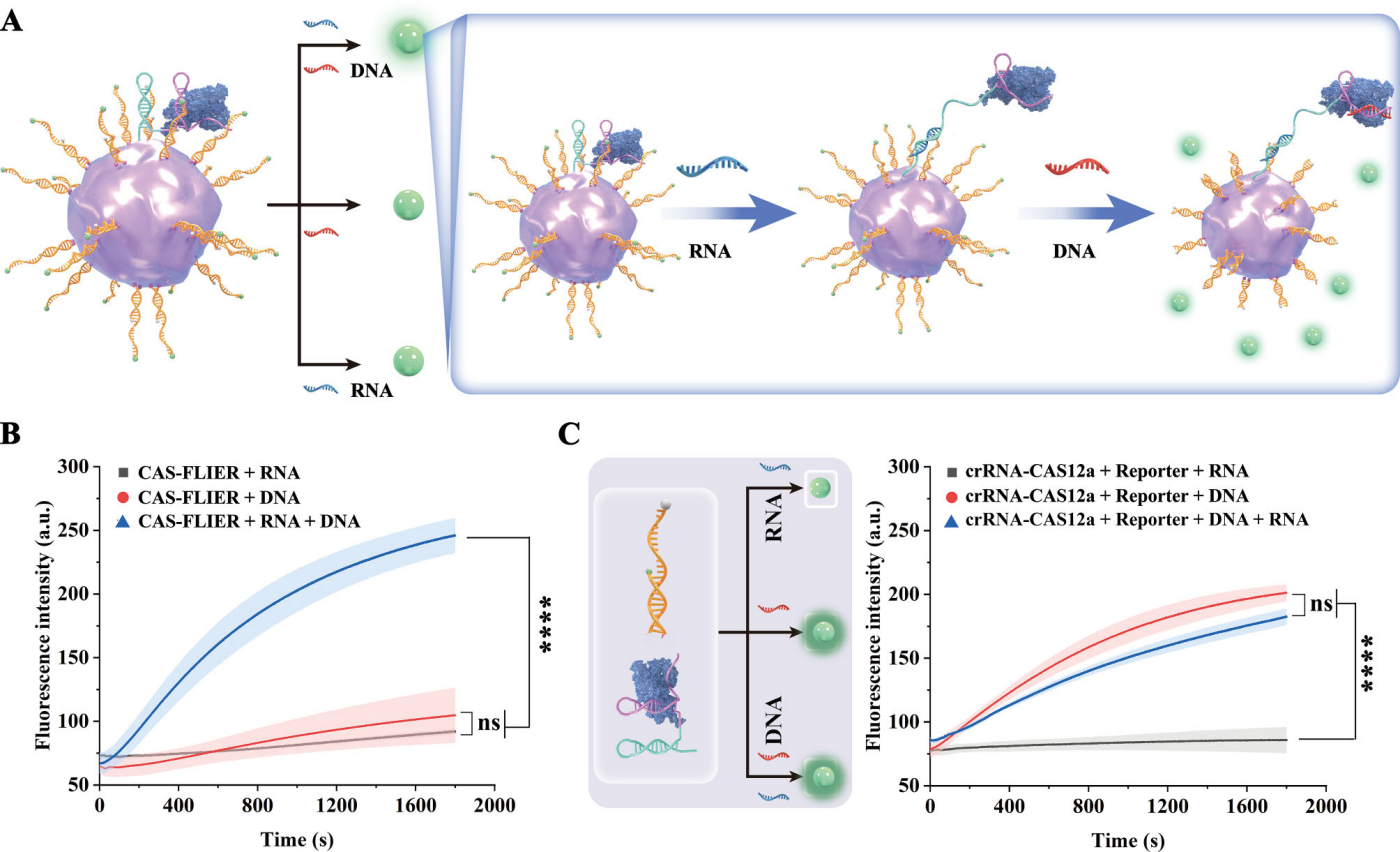
Synergistic activation of CAS-FLIER. (**A**) Schematic illustration of CAS-FLIER activation via the synergistic action of RNA and ssDNA. In the presence of both ssDNA and RNA, RNA unwinds the crRNA hairpin structure, followed by ssDNA binding to the crRNA spacer domain to activate CAS-FLIER. (**B**) Real-time fluorescence profiles of CAS-FLIER incubated with RNA alone, ssDNA alone, or both RNA and ssDNA. CAS-FLIER is activated exclusively in the presence of both ssDNA and RNA. (**C**) Schematic illustration (left) and real-time fluorescence profiles (right) of the freely dispersed CRISPR/Cas12a system incubated with RNA alone, ssDNA alone, or both RNA and ssDNA. Cas12a is activated in the presence of ssDNA regardless of RNA. RNA is 1 nM, and ssDNA is 1 nM (*n* = 3, mean ± s.d.). Reporter length: 38 nt. Statistical significance was determined by two-tailed unpaired Student’s t-test: ns, nonsignificant; ****P < 0.0001.

To test this hypothesis, we designed a hairpin-structured crRNA to constrain Cas12a on the RBC membrane surface. As shown in Fig. 3A, synthetic target RNA binds to and unwinds the crRNA hairpin, thereby enabling Cas12a to undergo long-range movement on the membrane. Subsequent hybridization of synthetic target DNA to the crRNA spacer then activates Cas12a trans-cleavage activity, leading to fluorescence signal release. Incubation of CAS-FLIER with both target RNA and ssDNA resulted in robust Cas12a activation, as evidenced by a sustained increase in fluorescence signal (*P* < 0.0001). By contrast, negligible activation was observed under all other conditions (target RNA or ssDNA alone), with only minimal signal fluctuations detected (*P* > 0.05; Fig. 3B). In principle, the adaptable CAS-FLIER platform can also be synergistically activated by dual ssDNA targets, provided that one ssDNA species unwinds the crRNA hairpin structure. However, in freely dispersed CRISPR/Cas12a systems (consisting of unanchored crRNA–Cas12a complexes and reporters), gradual fluorescence signal increases were observed both in the presence of ssDNA alone and in ssDNA-RNA mixtures, with no statistically significant differences between these conditions (*P* > 0.05; Fig. 3C). Collectively, these results demonstrate that CAS-FLIER can be configured as a dual-input gating responsive system, a capability that cannot be achieved in freely dispersed CRISPR/Cas12a systems. This design thus holds potential for the direct, simultaneous detection of two distinct target nucleic acids in a single assay.

### CAS-FLIER for HIV RNA detection

Timely diagnosis and antiretroviral therapy of human immunodeficiency virus (HIV) are crucial for reducing HIV transmission and mortality [50]. HIV RNA testing offers a shorter window period and higher diagnostic yield for acute HIV infection, making it more amenable to the early diagnosis of HIV infection [51, 52]. Quantitative reverse transcription-polymerase chain reaction (qRT-PCR) is the gold standard for HIV RNA detection, but requires skilled personnel to perform complex operations over extended periods in large clinical laboratories, which restricts its utility for POC diagnosis of HIV. Given CAS-FLIER’s superior capacity to modulate Cas12a trans-cleavage activity, we sought to develop a simple and rapid method for HIV RNA analysis based on this platform.

**Figure 4.**
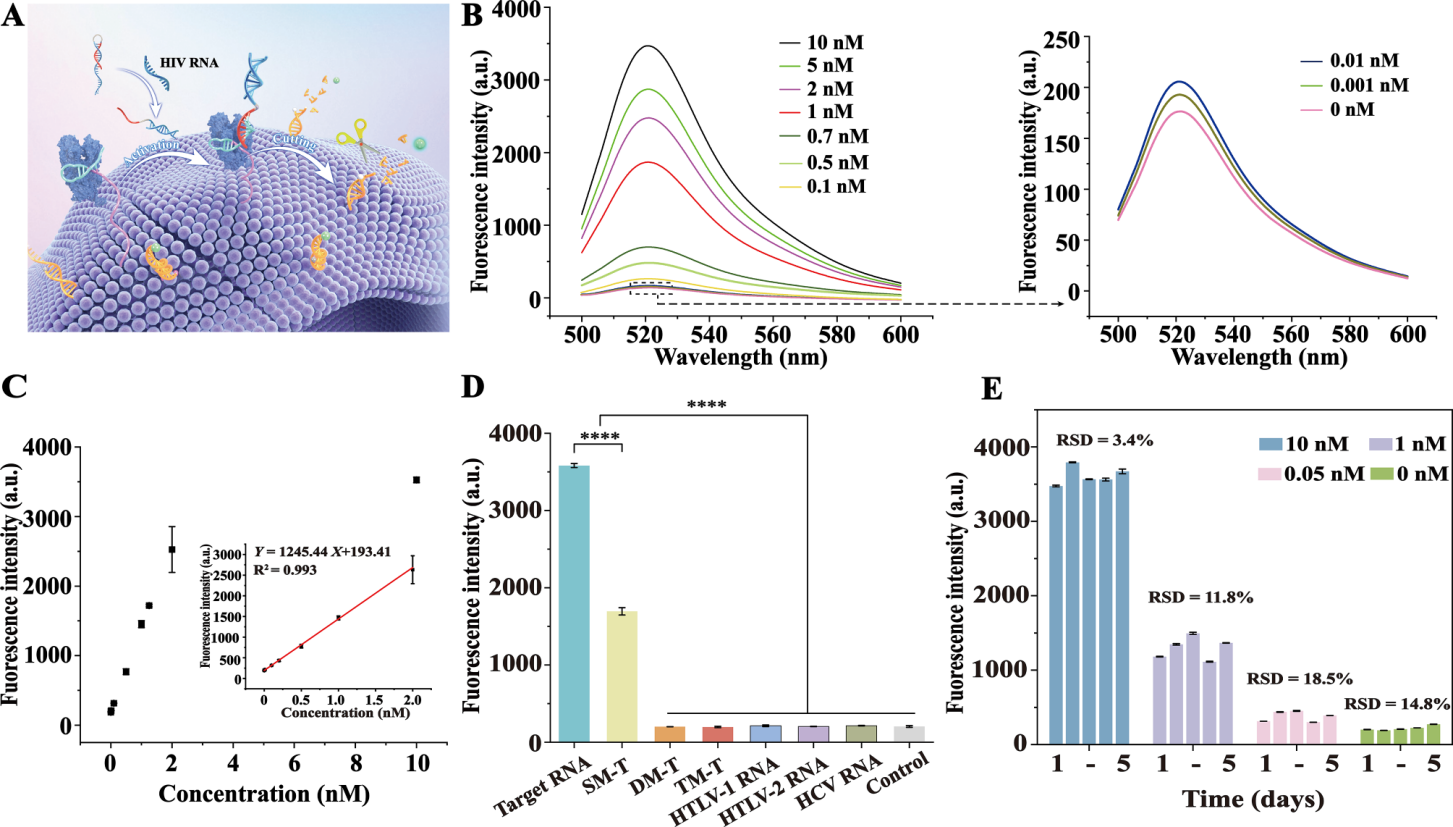
Performance characterization of the CAS-FLIER platform for synthetic HIV RNA detection. (**A**) Schematic illustration of the CAS-FLIER assay. HIV RNA hybridizes with a reverse-transcriptor to generate an ssDNA activator that triggers trans-cleavage of dual-labeled reporters by membrane-anchored Cas12a, thereby producing a fluorescent signal. (**B**) Fluorescence emission spectra of CAS-FLIER assays with increasing concentrations of synthetic HIV RNA (0.7–10 nM, left) and serial dilutions of HIV RNA (0–0.5 nM, right), showing concentration-dependent signal enhancement. (**C**) Fluorescence intensity (left) and corresponding calibration curve (inset) of CAS-FLIER for HIV RNA detection over 0–2 nM. The linear equation is Y = 1245.44X + 193.41 (*R*² = 0.993), where Y denotes fluorescence intensity, and X denotes target RNA concentration. (**D**) Specificity validation of CAS-FLIER. Fluorescence intensities for fully matched target RNA (10 nM), single-base mutant (SM-T; 10 nM), two-base mutant (DM-T; 10 nM), three-base mutant (TM-T; 10 nM), and blank control. ****P < 0.0001 (two-tailed unpaired Student’s t-test), relative to target RNA. (**E**) Stability assessment of CAS-FLIER. Fluorescence intensities of samples (10 nM, 1 nM, 0.05 nM HIV RNA, and blank control) over 5 days, with corresponding relative standard deviations (RSDs) indicating signal consistency. Data in (B–E) represent mean ± s.d. from three independent experiments. Error bars denote standard deviation.

Prior to HIV RNA detection, we first compared the reaction kinetics of CAS-FLIER with a reporter-localized CRISPR/Cas12a system and a dispersed CRISPR/Cas12a system using target DNA (Supplementary Fig. S9). The CAS-FLIER exhibited the highest catalytic efficiency (kcat/KM) of 4.0 × 104 M^−1^ s^−1^, which was 1.7-fold higher than that of the reporter-localized CRISPR/Cas12a system (kcat/KM = 2.3 × 104 M^−1^ s^−1^) and 10-fold higher than that of the dispersed CRISPR/Cas12a system (kcat/KM = 4.0 × 103 M^−1^ s^−1^). These results confirm that CAS-FLIER is well suited for constructing biosensing platforms for the detection of low-abundance targets. Owing to the low intrinsic sensitivity of Cas12a for direct RNA detection [53], we designed a reverse-transcriptor to convert HIV RNA recognition events into ssDNA activators (Fig. 4A). PAGE analysis confirmed that synthetic HIV RNA hybridizes with the DNA reverse-transcriptor, and the exposed ssDNA activators were further verified to trigger Cas12a-mediated reporter cleavage (evidenced by reporter degradation, Supplementary Fig. S10), thus validating the feasibility of CAS-FLIER for HIV RNA detection.

We next assessed the analytical sensitivity of CAS-FLIER for HIV RNA detection. As shown in Fig. 4B, the fluorescence signal increased with increasing target concentration from 1 pM to 10 nM, with a lowest detectable signal at 1.0 pM HIV RNA. A robust linear relationship was observed between fluorescence intensity and target RNA concentration over the range of 10 pM to 2 nM, with the fitted linear equation: *Y* = 1245.44X + 193.41 (*R*^2^ = 0.993), where Y denotes the fluorescence intensity, and X denotes target RNA concentration (Fig. 4C). The lowest detectable RNA concentration of CAS-FLIER (1.0 pM) represented a 100-fold improvement over the freely dispersed CRISPR/Cas12a system (100 pM), and a 10-fold improvement over the reporter-localized CRISPR/Cas12a system (10 pM; Supplementary Fig. S11). Collectively, these results establish the superior sensitivity of CAS-FLIER for quantitative HIV RNA detection.

To evaluate the specificity of CAS-FLIER, we synthesized three HIV RNA variants with different numbers of mismatched bases, as well as several retroviral RNAs with high sequence homology, and compared their fluorescence signals with those of fully matched target RNA. As shown in Fig. 4D, the fluorescence signal for fully matched target RNA was approximately 0.5-fold higher than that of the single-base mutant target RNA (SM-T; *P* < 0.0001), and about 18-fold higher than that of the two-base mutant (DM-T), three-base mutant (TM-T), as well as human T-cell lymphotropic virus type-1 (HTLV-1) RNA, HTLV-2 RNA, and HCV RNA (*P* < 0.0001). Notably, fluorescence intensities for DM–T, TM–T, HTLV–1 RNA, HTLV–2 RNA, and HCV RNA were nearly identical to those of the blank control. This high discrimination capacity confirms the excellent specificity of CAS-FLIER toward the target HIV RNA sequence.

We further evaluated the stability of CAS-FLIER for HIV RNA detection. As shown in Fig. 4E, fluorescence signals generated by target RNA at concentrations of 10, 1, 0.05, and 0 nM remained relatively stable over a 5-day incubation period. These findings verify the acceptable biostability of the CAS-FLIER. We additionally assessed the anti-interference capability of CAS-FLIER by testing target RNA spiked into 5% serum. Fluorescence intensities of serum-containing samples were comparable to control samples (target RNA in 2 × NEBuffer 2.1) with no statistically significant differences (*P* > 0.05; Supplementary Fig. S12). CAS-FLIER thus exhibits excellent tolerance to biological matrix interference, a critical attribute for clinical applicability. We next performed recovery experiments to validate the accuracy of CAS-FLIER for HIV RNA detection. As shown in Supplementary Table S2, the recoveries of synthetic HIV RNA spiked into healthy human serum at concentrations of 100, 200, 500 pM, and 1 nM ranged from 90.1 to 105.0%, with relative standard deviations (RSDs) of 2.0 to 4.7%. These data confirm the high detection accuracy of CAS-FLIER for HIV RNA in biological matrices.

### CAS-FLIER for HIV infection diagnosis

**Figure 5.**
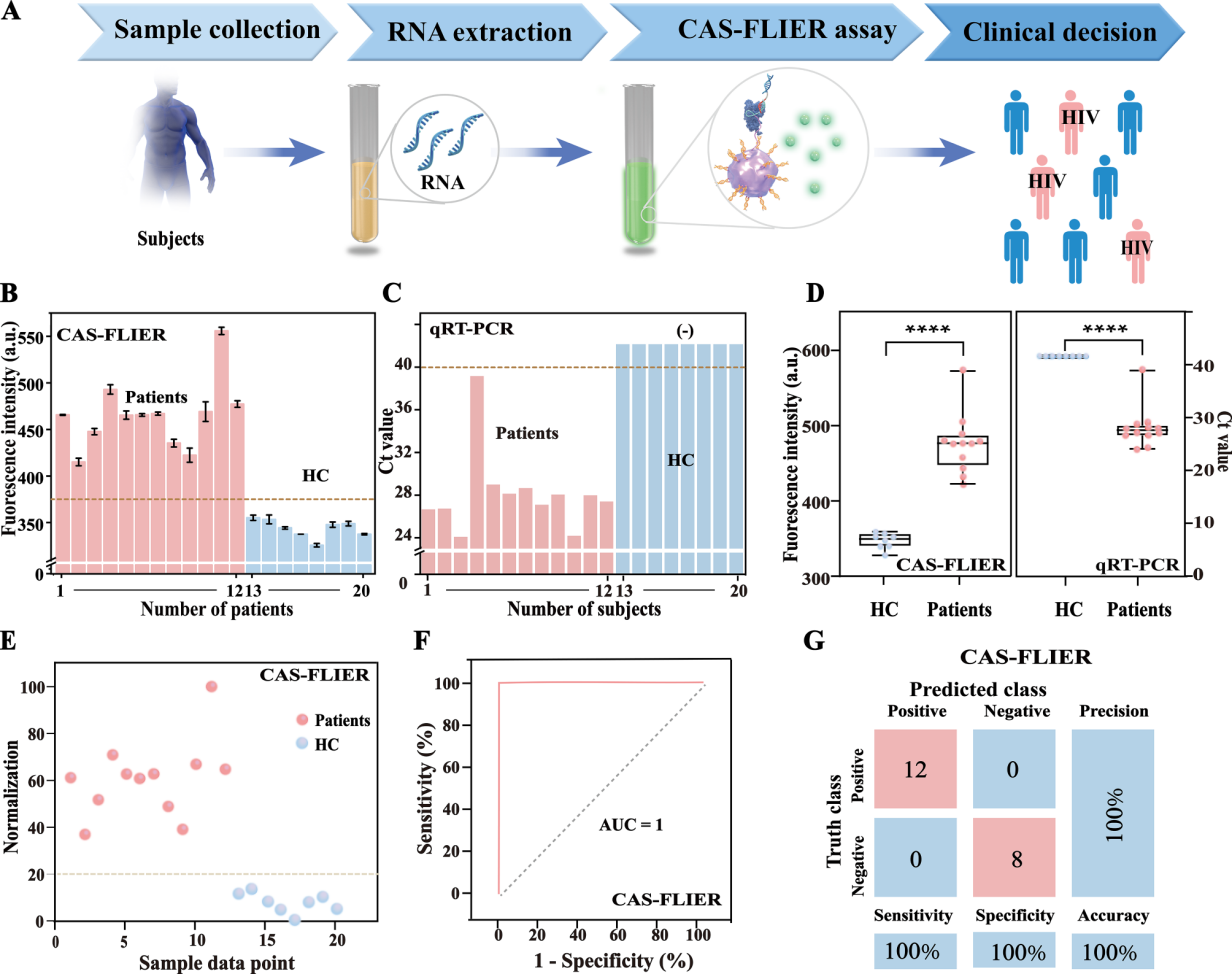
Clinical validation of CAS-FLIER for HIV infection diagnosis via HIV RNA detection. (**A**) Schematic workflow for HIV diagnosis, including patient sample collection, RNA extraction, CAS-FLIER assay, and clinical classification of HIV status. (**B**) Fluorescence intensities of CAS-FLIER assays for HIV-positive patients and healthy controls (HC). The dashed line indicates the threshold for positive detection. (**C**) qRT-PCR Ct values for patient and HC samples. Ct < 40 is defined as positive. (**D**) Statistical comparison of fluorescence intensities (CAS-FLIER) and Ct values (qRT-PCR) between patients and HC (****P < 0.0001, two-tailed unpaired Student’s t-test). (**E**) Normalized CAS-FLIER fluorescence intensities for individual patient and HC samples, obtained by normalizing raw fluorescence signals to the range of 0–100 relative to maximum positive and minimum background signals. (**F**) Receiver operating characteristic (ROC) curve of CAS-FLIER for distinguishing patients from HC. The area under the curve (AUC) is 100.0%. (**G**) Confusion matrix of CAS-FLIER for clinical sample classification, demonstrating 100.0% sensitivity, specificity, and accuracy (12 positive samples, 8 negative samples). Data in (B–E) are presented as mean ± s.d. from three independent experiments.

Given the superior analytical performance of CAS-FLIER, we next explored its clinical utility for diagnosing HIV infection. We collected 20 serum samples (12 from HIV-positive patients, 8 from healthy donors) from Chongqing Hospital of Traditional Chinese Medicine. RNA was extracted from these samples, analyzed via the CAS-FLIER assay, and used to guide clinical classification (Fig. 5A). In addition, the extracted RNA was analyzed by qRT-PCR and the conventional CRISPR-Cas12a system.

As shown in Fig. 5B, CAS-FLIER generated significantly higher fluorescence intensities in HIV-positive patients than in healthy controls (HC). These results aligned with qRT-PCR outcomes: HIV patients exhibited low Ct values (a Ct < 40 was defined as positive), whereas HC samples showed no detectable signal (Fig. 5C). As shown in Fig. 5D, statistical analysis confirmed that CAS-FLIER effectively discriminated HIV patients from HC with highly significant differences (*P* < 0.0001), which was consistent with qRT-PCR (*P* < 0.0001). In contrast, the conventional CRISPR-Cas12a system exhibited poor diagnostic performance for HIV patients, due to its relatively low sensitivity (Supplementary Fig. S13). Furthermore, HIV patients and HC samples were completely separated based on normalized fluorescence signals (Fig. 5E). A receiver operating characteristic (ROC) curve for CAS-FLIER yielded an area under the curve (AUC) of 100.0% (Fig. 5F), indicating perfect diagnostic performance for identifying HIV-infected individuals. A confusion matrix further confirmed that CAS-FLIER correctly classified all 12 positive samples and 8 negative samples, achieving 100.0% sensitivity, specificity, and accuracy (Fig. 5G). Collectively, these results demonstrate that CAS-FLIER enables accurate diagnosis of HIV infection.

### CAS-FLIER combines with lateral flow strips for POC diagnosis of HIV infection

**Figure 6.**
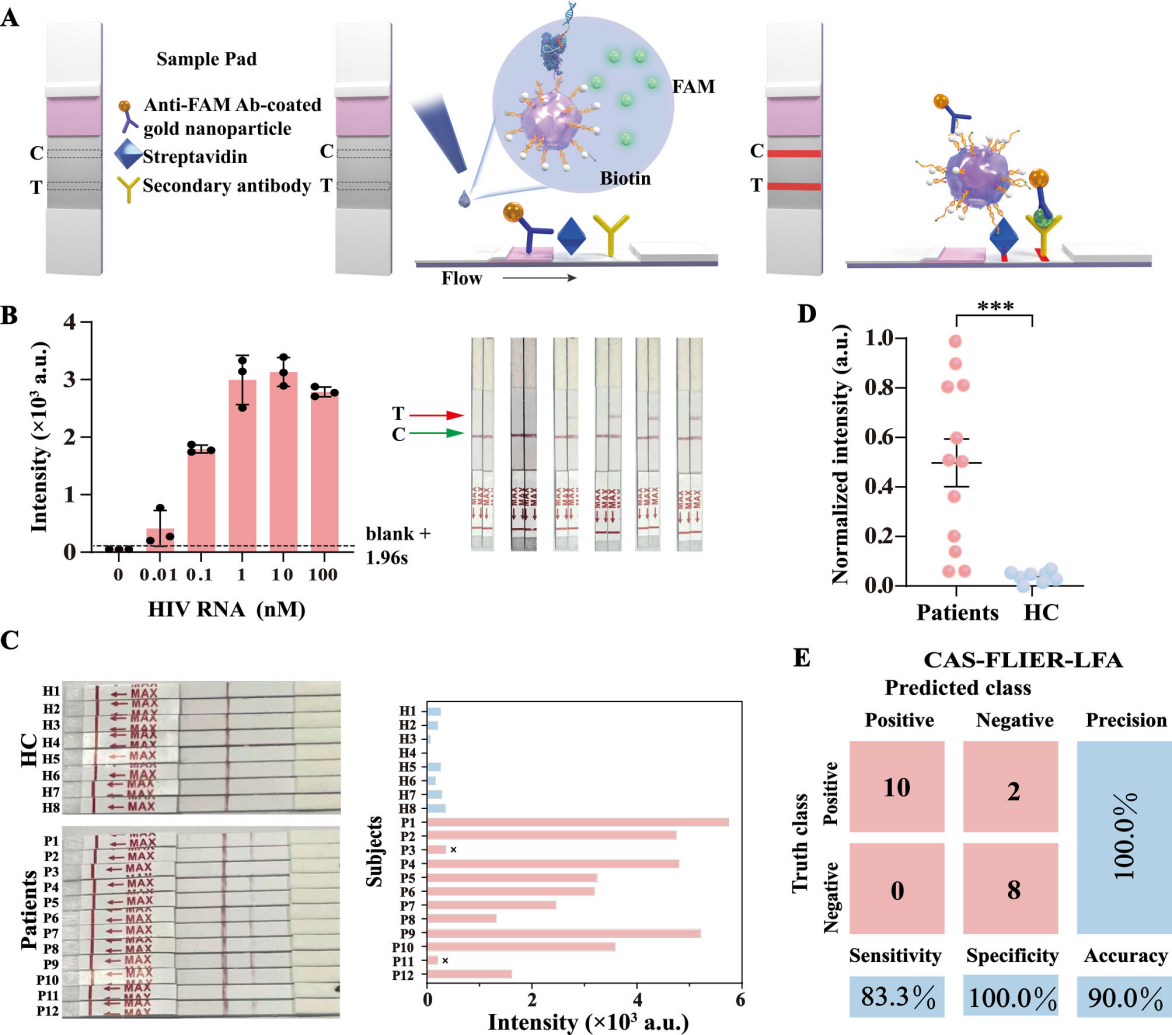
Integration of CAS-FLIER with a lateral flow assay for POC diagnosis of HIV infection. (**A**) Schematic illustration of the CAS-FLIER-LFA platform. The strip is functionalized with anti-FAM Ab-conjugated gold nanoparticles, a streptavidin-coated control band, and a secondary Ab-coated test band. (**B**) Representative photos (left) and corresponding bar graphs (right) showing test band intensities of CAS-FLIER-LFA for synthetic HIV RNA detection at concentrations ranging from 0 to 100 nM. The dashed line indicates detection of a statistically significant signal relative to the blank. Error bars represent standard deviation (s.d., *n* = 3). (**C**) Photos (left) and qualitative visual readouts (right) of CAS-FLIER-LFA for HIV-positive patient samples and HC samples. (**D**) Scatter dot plots of qualitative visual signals from CAS-FLIER-LFA for HIV-positive patient samples (*n* = 12) and HC samples (*n* = 8) (***P < 0.001, two-tailed unpaired Student’s t-test). (**E**) Confusion matrix analysis of CAS-FLIER-LFA for clinical sample classification, demonstrating a sensitivity of 83.3%, specificity of 100.0%, and accuracy of 90.0% (12 positive, 8 negative samples).

To expand the applicability of CAS-FLIER for convenient, instrument-free diagnosis of HIV infection, we integrated this platform with a lateral flow strip assay (CAS-FLIER-LFA). As shown in Fig. 6A, the lateral flow strip was pre-loaded with anti-FAM antibody (Ab)-coated gold nanoparticles, streptavidin immobilized on the control line, and a secondary Ab immobilized on the test line. To adapt CAS-FLIER for compatibility with the lateral flow format, membrane-anchored double-stranded reporters were modified with biotin at the 5ʹ-end of one strand and a FAM molecule at the 3ʹ-end of its complementary strand. Upon application of CAS-FLIER-incubated samples to the strip, anti-FAM Ab-coated gold nanoparticles captured FAM molecules; intact reporters were immobilized on the streptavidin-coated control line via biotin-streptavidin interaction to generate a visible red control band, whereas Cas12a-mediated cleavage of reporters released FAM molecules that were subsequently captured by the secondary Ab on the test line, yielding a red positive band. In the absence of HIV RNA, Cas12a trans-cleavage activity was not activated, precluding FAM release and resulting in an undetectable test line with no visible colour development.

We first assessed the analytical sensitivity of the CAS-FLIER-LFA using synthetic HIV RNA. As shown in Fig. 6B, the visual intensity of the test line increased with increasing target HIV RNA concentrations from 0 to 10 nM, with a lowest detectable RNA concentration of 10 pM. We subsequently validated the clinical practicability of CAS-FLIER-LFA using the same cohort of HIV-positive patients and HC serum samples. As shown in Fig. 6C and D, CAS-FLIER-LFA enabled visual, qualitative identification of 10 out of 12 HIV-positive samples, with no false-positive signals detected in HC samples across all assays. As shown in Fig. 6E, confusion matrix analysis confirmed that CAS-FLIER-LFA effectively identified HIV infection with a sensitivity of 83.3% (10/12), specificity of 100.0% (8/8), overall accuracy of 90.0% (18/20), and precision of 100.0% (10/10). Collectively, these results demonstrate that CAS-FLIER-LFA holds substantial potential for POC diagnosis of HIV infection via HIV RNA analysis.

## Conclusion

In summary, we achieve flexible, highly scalable regulation of Cas12a trans-cleavage activity via a spatial confinement effect, exemplified by the spatial confinement of Cas12a on fluid lipid membranes to form CAS-FLIER. We identify three key parameters—crRNA extension direction and length, and double-stranded reporter length—that strongly regulate Cas12a activity in the CAS-FLIER, in contrast to previously reported freely dispersed Cas12a systems that rely on intricate crRNA engineering or specialized chemical modifications. We demonstrate that the trans-cleavage activity of Cas12a in CAS-FLIER can be programmably modulated over a ∼70-fold dynamic range (from 5.6 × 10² to 4.0 × 10^4^ M^−1^ s^−1^). In principle, rational selection of crRNA length, crRNA extension direction, and reporter length enables the bespoke tuning of Cas12a activity for diverse applications, including gene editing and molecular diagnostics. This spatial confinement-based regulatory approach is readily translatable to other Cas nucleases, enabling the fine-tuning of their catalytic activity for broad biotechnological applications.

Building on these findings, we designed a hairpin-structured crRNA that acts as a molecular switch to govern Cas12a mobility, thereby constructing a dual-input gated responsive system. In principle, this dual-input gating can be triggered by RNA and DNA, or ssDNA and DNA, thus expanding the functional scope of Cas12a-based biosensing platforms. Furthermore, upon integration with a DNA reverse-transcriptor, CAS-FLIER achieves highly sensitive and specific detection of HIV RNA, with 100.0% sensitivity, specificity, and accuracy for the diagnosis of HIV infection in a clinical cohort comprising 12 HIV-positive patients and 8 healthy controls. When combined with a lateral flow strip assay, CAS-FLIER enables effective POC diagnosis of HIV infection, with a sensitivity of 83.3%, specificity of 100.0%, and overall accuracy of 90.0%. Collectively, this work establishes a versatile strategy for simple, programmable, and scalable modulation of Cas12a trans-cleavage activity, opens a new avenue for synergistic activation of Cas12a, and provides a rapid POC diagnostic tool for viral infections.

## Supplementary Material

gkag414_Supplemental_File

## Data Availability

All data supporting the findings of this study are available within the article and its supplementary information or will be made available from the authors upon request.
