# Supplementary material for: Flexible regulation of CRISPR/Cas12a activity by spatial confinement effect
Source: Nucleic Acids Res. 2026 May 4;54(8):gkag414. doi: 10.1093/nar/gkag414 (PMC13136891; doi:10.1093/nar/gkag414)
Supplement: gkag414_Supplemental_File [file gkag414_supplemental_file.docx]

**Supporting Information**

**Flexible regulation of CRISPR/Cas12a activity by spatial confinement effect**

Huan Yang,^†,1^ Bo Shen,^†,2^ Yuwei Wang,^†,2^ Jianxiong Liu,^†,3^ Fangzhu Zhou,^†,2^ Min Liu,^1^ Jie Li,^2^ Jinjin Fan,^4^ Shijia Ding,^3^ Jinlin Guo,^1^ Juan Zhang,*^,1,2^ Xinmin Li,*^,2^

^1^ College of Medical Technology, Chengdu University of Traditional Chinese Medicine, Chengdu 611137, China

^2^ Department of Laboratory Medicine, Chongqing Hospital of Traditional Chinese Medicine, Chongqing 400021, China

^3^ Key Laboratory of Clinical Laboratory Diagnostics (Ministry of Education), College of Laboratory Medicine, Chongqing Medical University, Chongqing 400016, China

^4^ Key Lab for Special Functional Materials of Ministry of Education, Henan University, Kaifeng 475004, China

*To whom correspondence should be addressed. Email: lixinmin@cdutcm.edu.cn

Correspondence may also be addressed to Juan Zhang. Email: zhangjuan@cdutcm.edu.cn

†The authors wish it to be known that, in their opinion, the first five authors should be regarded as Joint First Authors.

**Table of Contents**

**Figure S1 1**

**Figure S2 2**

**Figure S3 3**

**Figure S4 4**

**Figure S5 5**

**Figure S6 5**

**Figure S7 6**

**Figure S8 7**

**Figure S9 7**

**Figure S10 8**

**Figure S11 9**

**Figure S12 10**

**Figure S13 10**

**Table S1 11**

**Table S2 12**


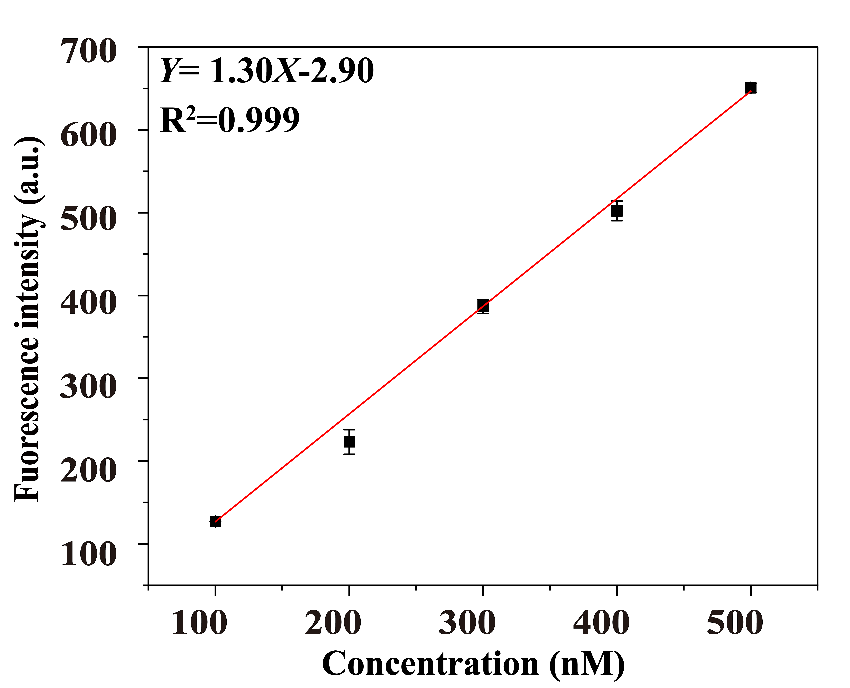


**Figure S1.** Linear standard curve of fluorescence intensity vs cholesterol- and FAM-labeled reporter concentration. Serial concentrations of cholesterol- and FAM-dual-labeled reporter were subjected to fluorescence intensity measurement for calibration curve construction, with the linear regression equation: *Y* = 1.30*X* − 2.90 (where *Y* represents the measured fluorescence intensity and *X* represents the concentration of the reporter probe in nM). This curve was applied for the quantification of unbound reporter in the supernatant.

**
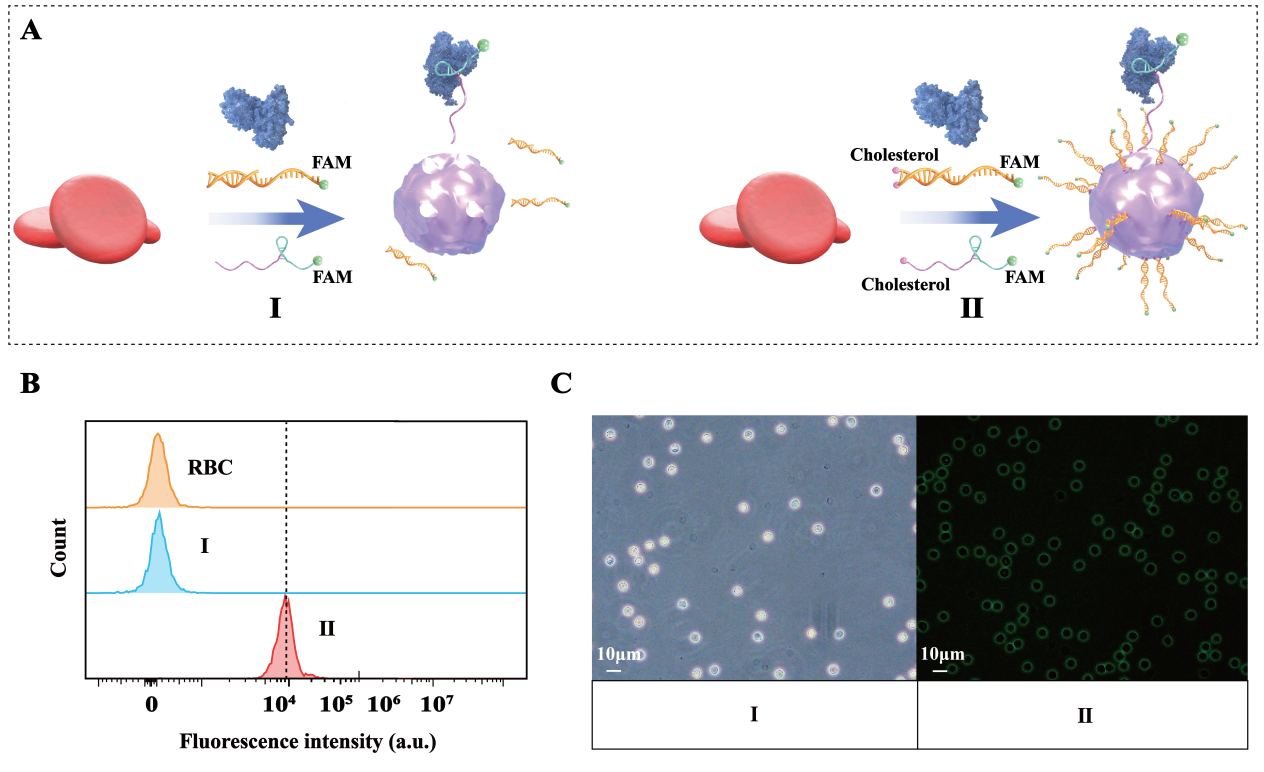
Figure S2.** Evaluation of the binding of cholesterol-modified strands to red blood cell (RBC) membranes. (A) Schematic of interactions between FAM-modified nucleic acid strands (crRNA and double-stranded reporters) and red RBC membranes (Ⅰ), and between FAM- and cholesterol-dual-modified strands and RBC membranes (Ⅱ). (B) Flow cytometry (FCM) analysis showing significantly higher fluorescence signals in Ⅱ relative to Ⅰ and untreated RBCs, and fluorescence signals in Ⅰ were nearly identical to those in untreated RBCs. (C) Representative confocal fluorescence images in Ⅰ or Ⅱ. After incubation with nucleic acid strands, all RBC samples were thoroughly washed with PBS buffer by centrifugation at 3500 rpm for 5 min to remove unbound excess nucleic acids. Thus, these results confirm that crRNA and double-stranded reporters binding to RBC membranes occur via cholesterol-lipid bilayer interactions rather than non-specific adsorption.

**
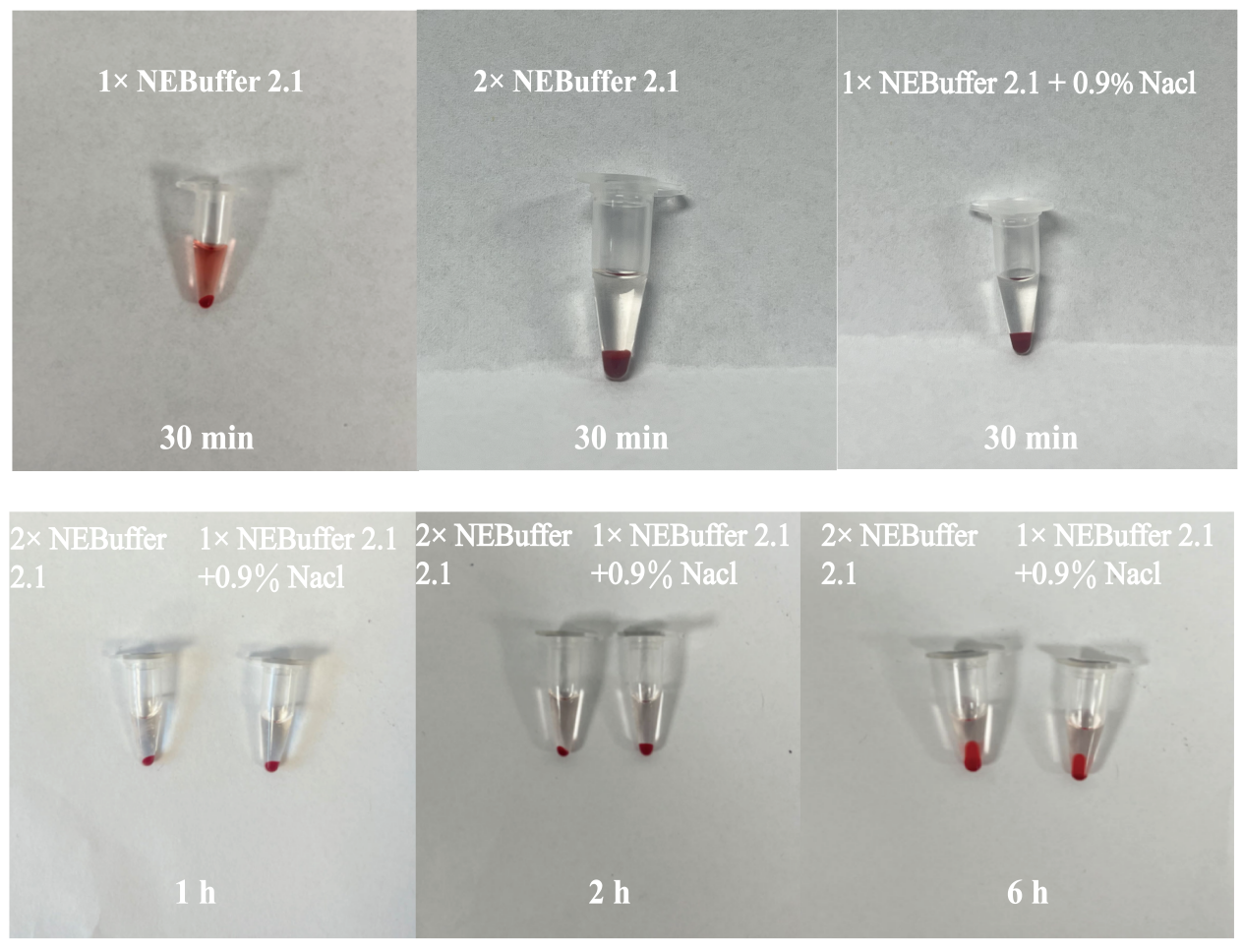
**

**Figure S3.** Representative photographs of CAS-FLIER in three distinct reaction buffers incubated at 37 ℃ for different durations. CAS-FLIER in 1× NEBuffer 2.1 underwent hemolysis, whereas no observable hemolysis was exhibited by CAS-FLIER in 2× NEBuffer 2.1 or 1× NEBuffer 2.1 supplemented with 0.9% NaCl, even after 2 h of incubation at 37 ℃.


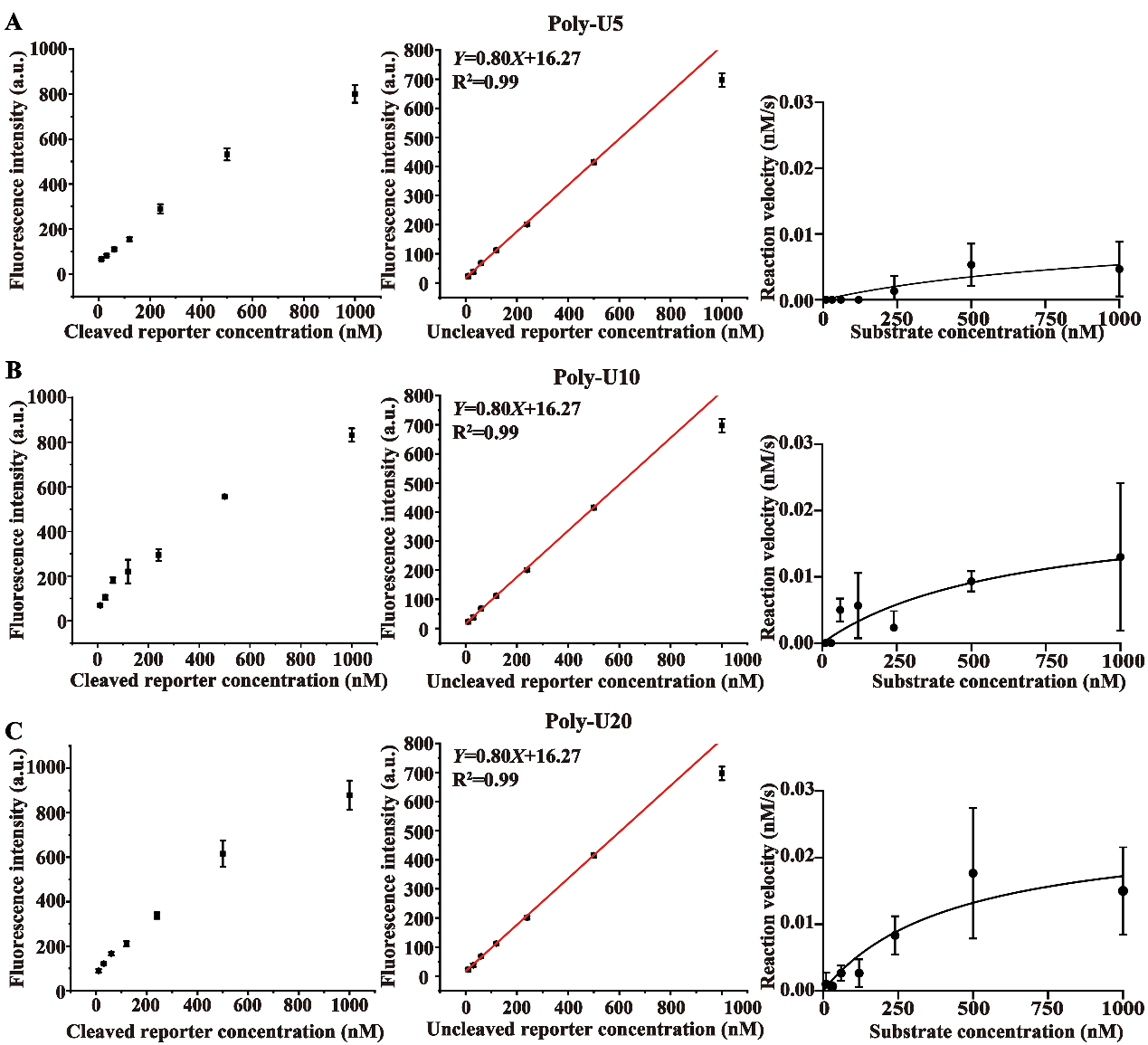


**Figure S4.** Michaelis–Menten kinetics of Cas12a within CAS-FLIER for *trans*-cleavage of 28-nt reporters, using crRNAs extended at the 5’-end with different lengths of polyuracil (poly-U) bases. (A) *Trans*-cleavage activity of Cas12a with 5’-poly-U5 crRNA. (B) *Trans*-cleavage activity of Cas12a with 5’-poly-U10 crRNA. (C) *Trans*-cleavage activity of Cas12a with 5’-poly-U20 crRNA. Error bars represent mean ± standard deviation (s.d.) from three independent experiments.

**
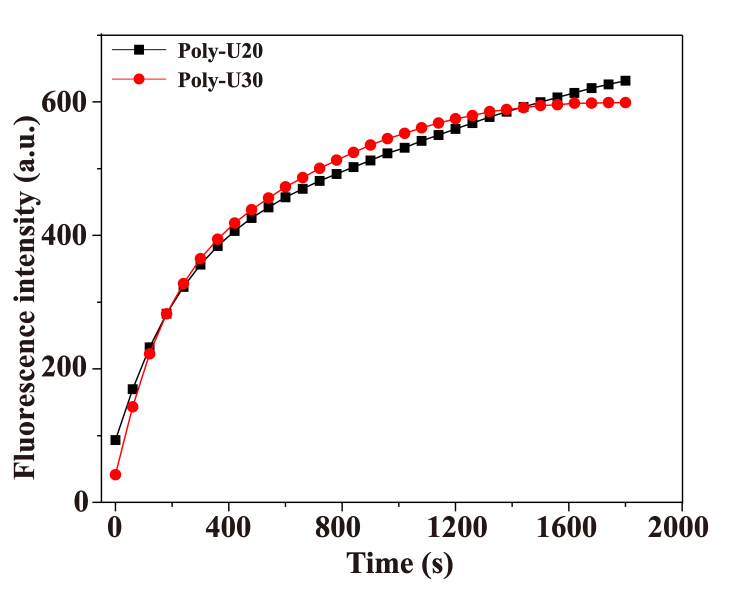
**

**Figure S5.** Real-time fluorescence profiles of Cas12a within CAS-FLIER for *trans*-cleavage of 28-nt reporters using 5’-Poly-U20 and 5’-Poly-U30 crRNA. Cas12a *trans*-cleavage activity was nearly identical between 5’-Poly-U20 and 5’-Poly-U30 crRNA.


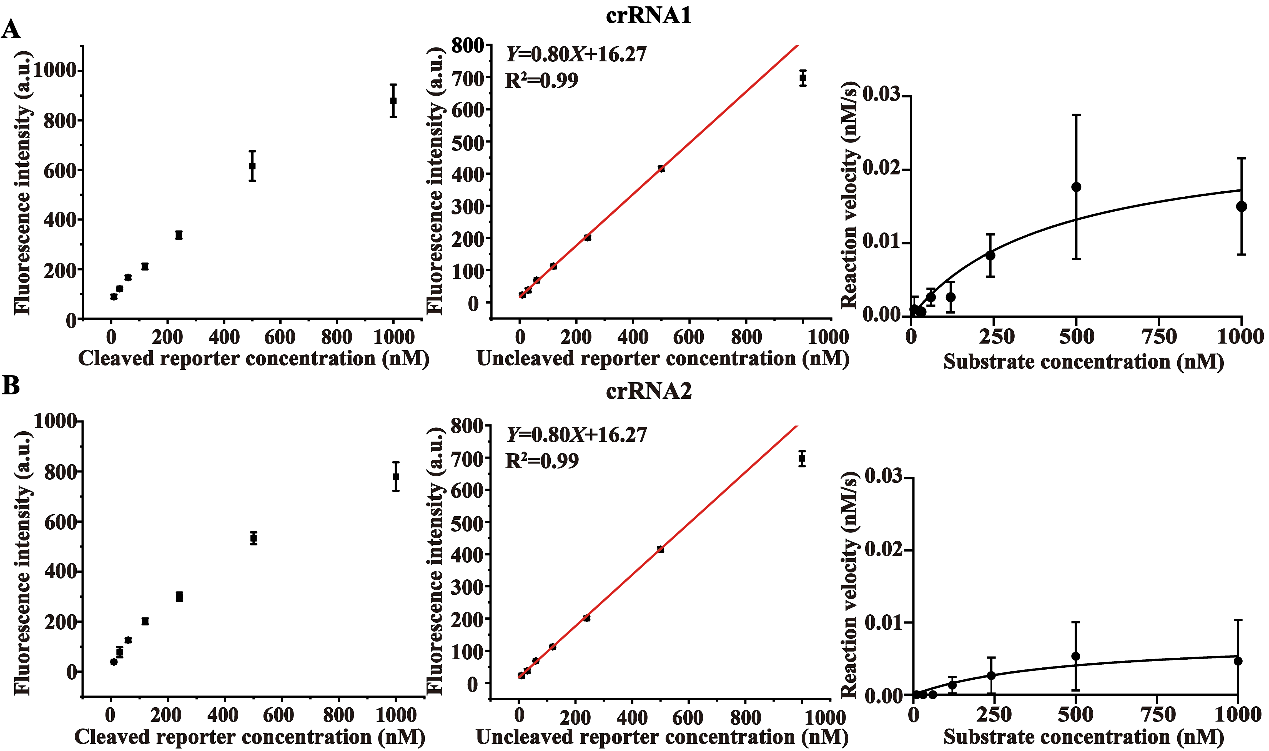


**Figure S6.** Michaelis–Menten kinetics of Cas12a within CAS-FLIER for *trans*-cleavage of 28-nt reporters, using crRNAs extended in different directions. (A) *Trans*-cleavage activity of Cas12a with 5’-poly-U20 crRNA (crRNA1; 20 consecutive uracils at the crRNA 5’-end). (B) *Trans*-cleavage activity of Cas12a with 3’-poly-U20 crRNA (crRNA2; 20 consecutive uracils at the crRNA 3’-end). Error bars represent mean ± standard deviation (s.d.) from three independent experiments.


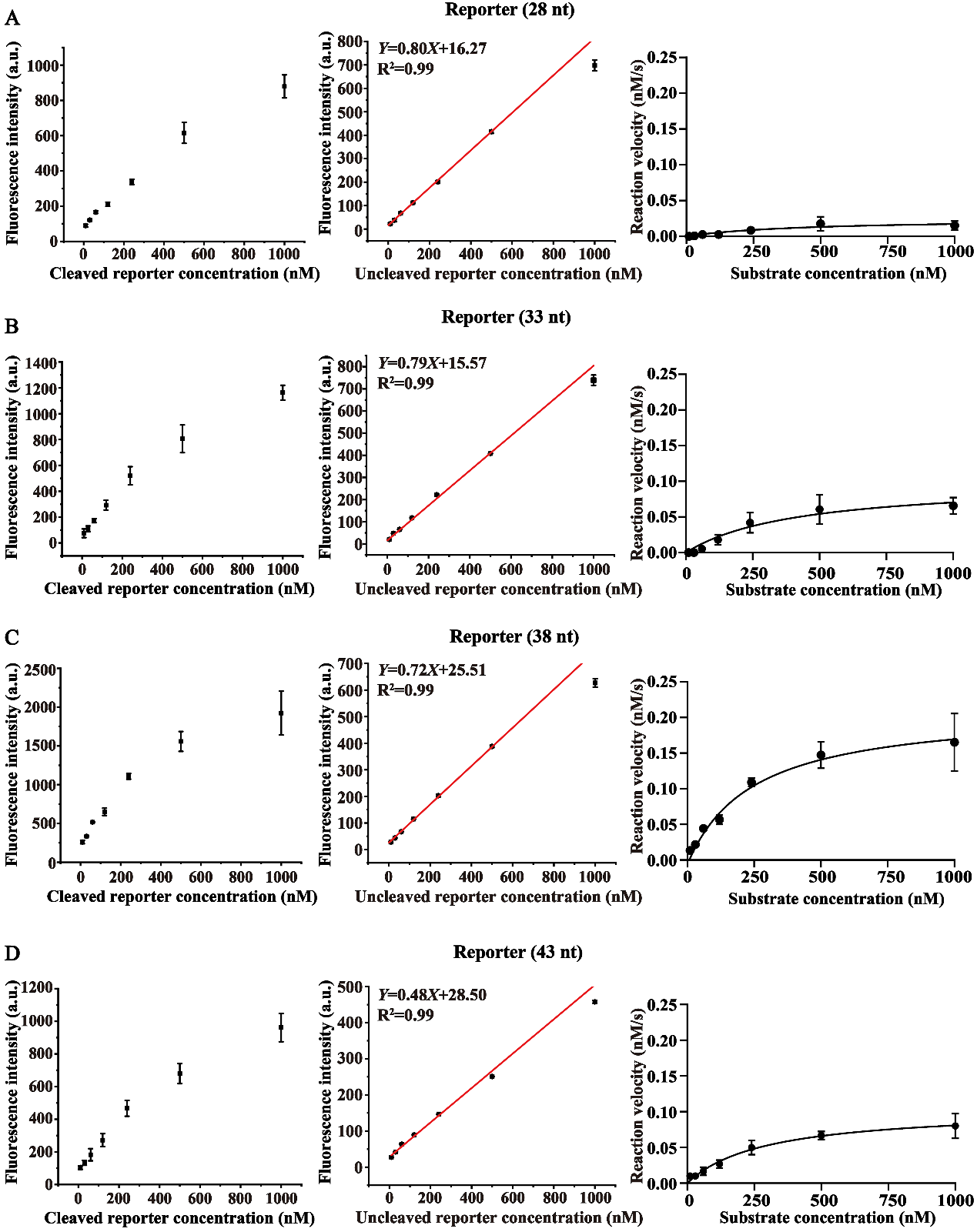


**Figure S7.** Michaelis–Menten kinetics of Cas12a within CAS-FLIER for *trans*-cleavage of reporters with different lengths. (A) *Trans*-cleavage of 28-nt reporters. (B) *Trans*-cleavage of 33-nt reporters. (C) *Trans*-cleavage of 38-nt reporters. (D) *Trans*-cleavage of 43-nt reporters. Error bars represent mean ± standard deviation (s.d.) from three independent experiments.


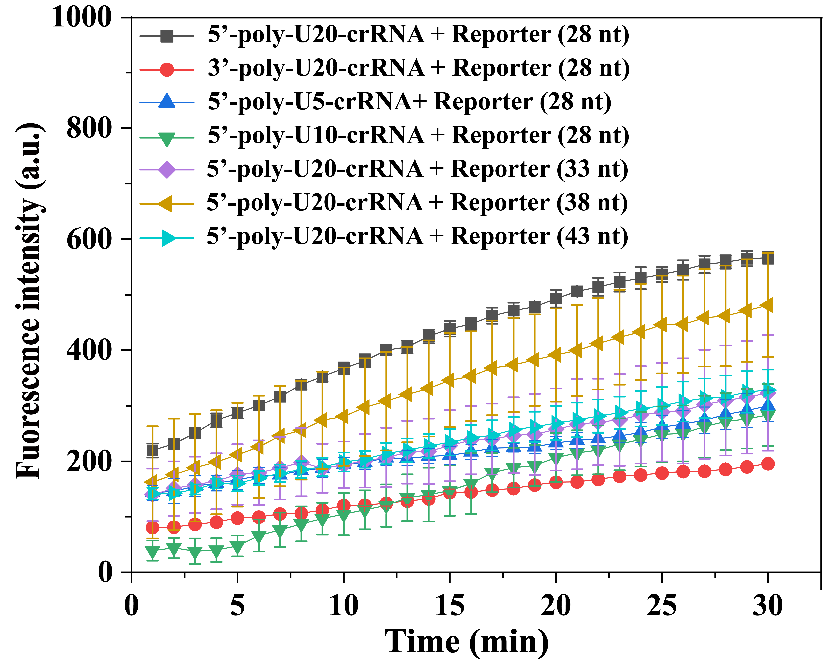


**Figure S8.** Real-time fluorescence profiles of the freely dispersed CRISPR/Cas12a system under different experimental conditions (4 nM crRNA, 4 nM Cas12a, 200 nM reporter, 10 nM target DNA). Fluorescence signals were recorded every 30 s using a BioTek SYNERGY H1 microplate reader. Error bars represent mean ± s.d. from three independent experiments.

**
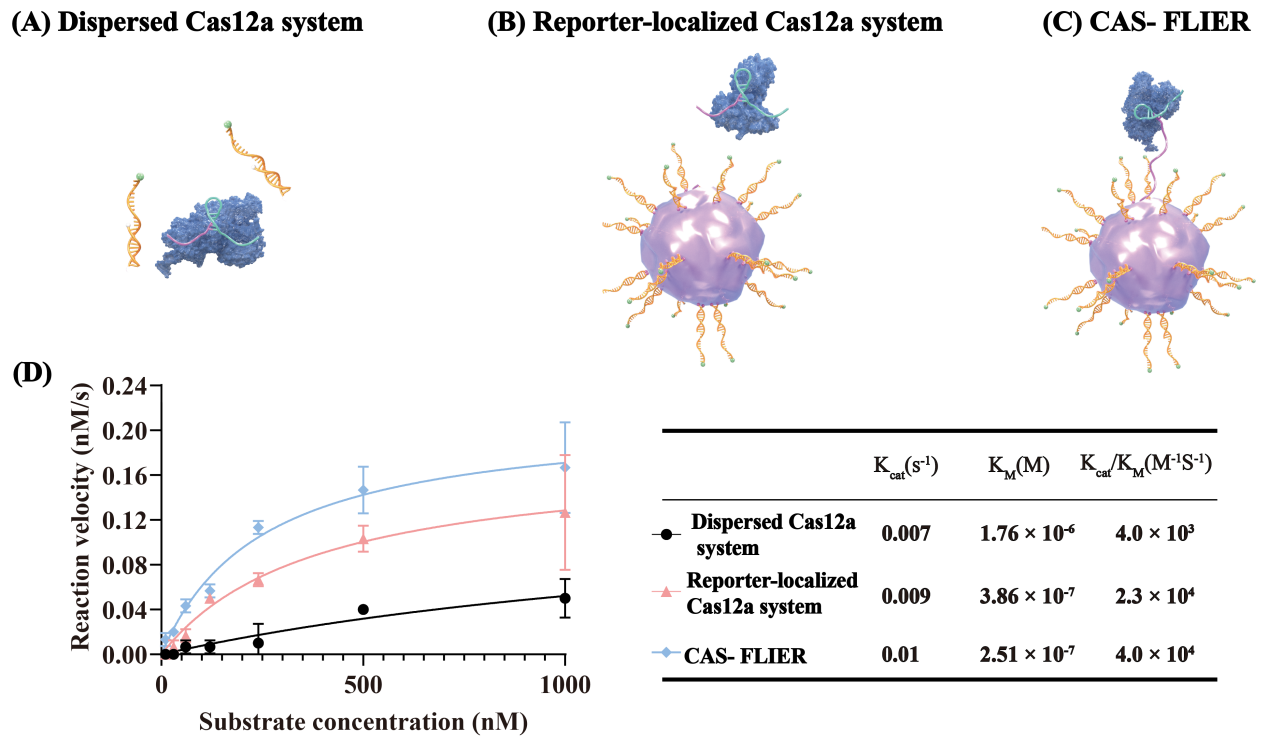
**

**Figure S9.** Comparison of reaction kinetics for freely dispersed CRISPR/Cas12a (A), reporter-localized CRISPR/Cas12a (B), and CAS-FLIER (C) with 10 nM target DNA. (D) Reaction velocity versus substrate concentration curves (left) and quantitative analysis of trans-cleavage activity (right) for the three systems. CAS-FLIER exhibited the highest catalytic efficiency (*k*_cat_/*K*_M_) of 4.0 × 10^4^ M⁻¹ s⁻¹. Error bars represent mean ± standard deviation (s.d.) from three independent experiments.

**
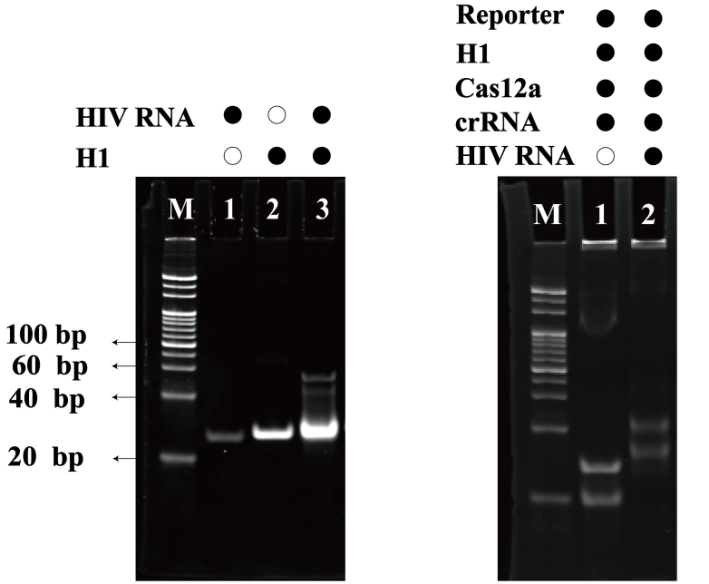
**

**Figure S10.** Polyacrylamide gel electrophoresis (PAGE) validation of HIV RNA-H1 hybridization and Cas12a-mediated reporter cleavage. Left: Schematic (top) and PAGE analysis (bottom) of HIV RNA-H1 hybridization: lane 1 (HIV RNA), lane 2 (H1), lane 3 (HIV RNA + H1). The shifted band in lane 3 confirms HIV RNA-H1 complex formation. Right: Schematic (top) and PAGE analysis (bottom) of Cas12a-mediated reporter cleavage: lane 1 (no HIV RNA), lane 2 (intact system). Lane M: 20 bp DNA ladder.

**
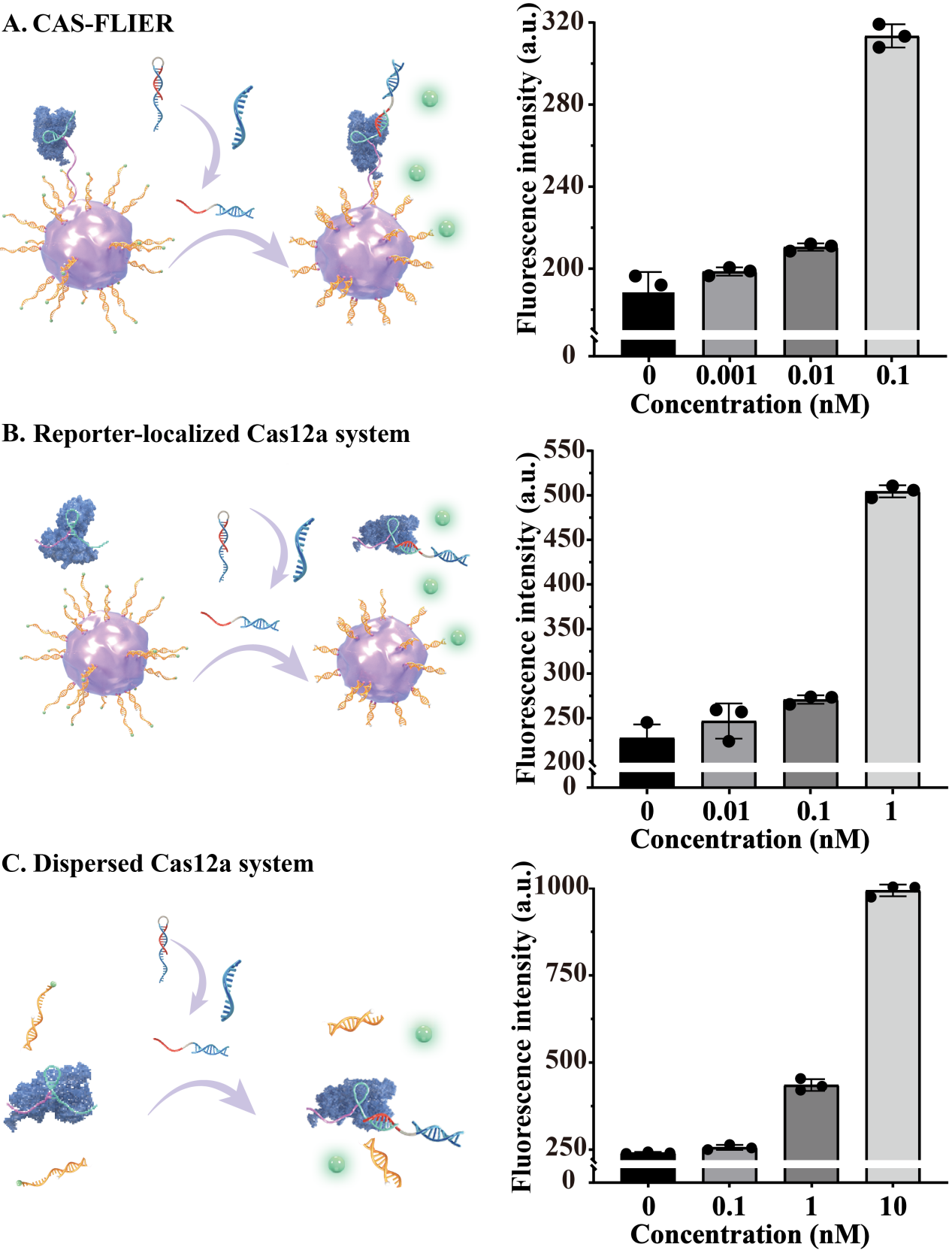
Figure S11.** Sensitivity comparison of CAS-FLIER, reporter-localized Cas12a system, and dispersed Cas12a system. Schematic illustrations (left) and corresponding fluorescence intensity dose-response curves (right) for (A) CAS-FLIER, (B) reporter-localized Cas12a system, and (C) freely dispersed Cas12a system. CAS-FLIER achieved a lowest detectable concentration of 1.0 pM for target RNA, representing a 10-fold improvement over the reporter-localized system (10 pM) and a 100-fold improvement over the dispersed system (100 pM). Error bars represent mean ± s.d. from three independent experiments.


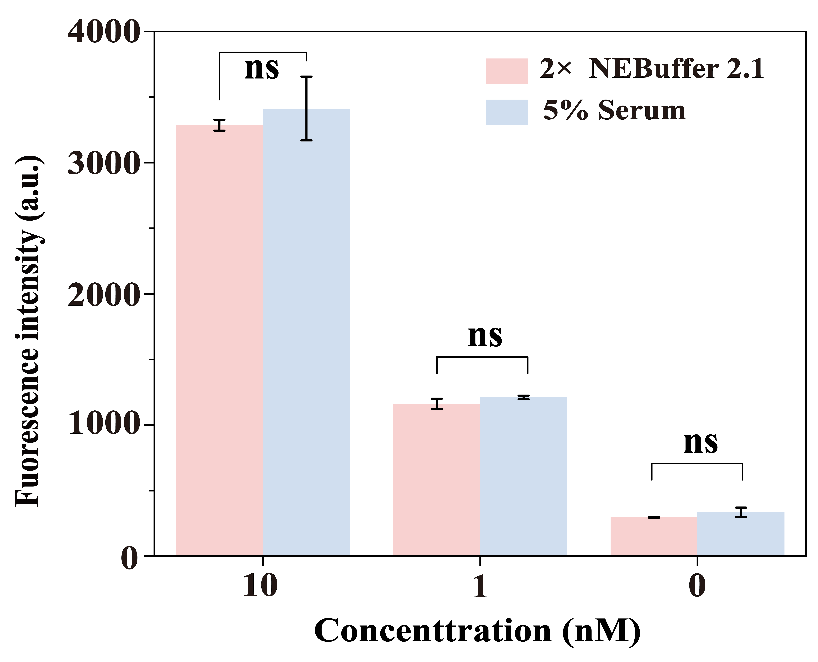


**Figure S12.** Fluorescence signals of the CAS-FLIER in response to 10 nM, 1 nM, and 0 nM target RNA in 2× NEBuffer 2.1 and 5% serum samples. Error bars represent mean ± s.d. from three independent experiments.


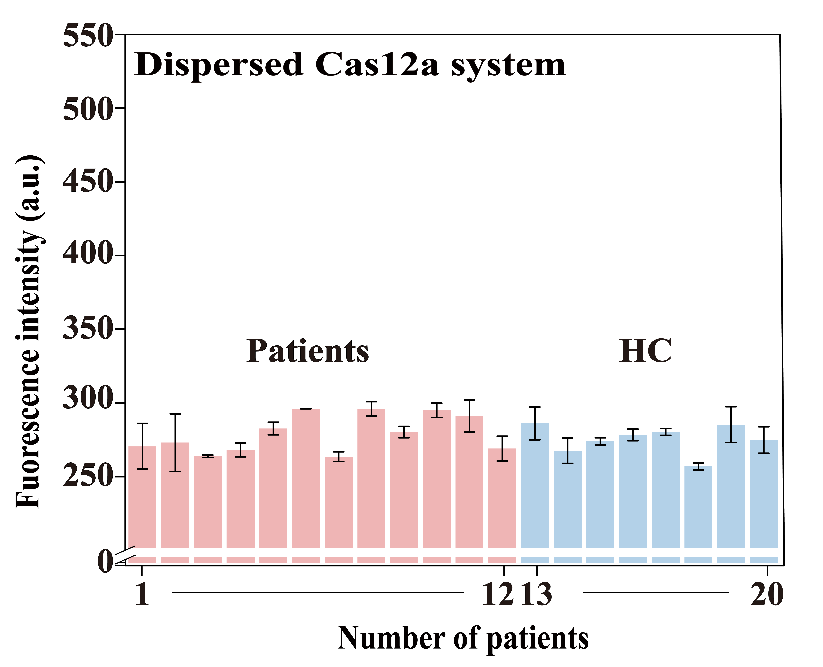


**Figure S13.** Fluorescence intensities obtained using conventional CRISPR-Cas12a system assays for HIV-positive patients (n = 12) and healthy controls (n = 8). Data are presented as mean ± s.d. from three independent experiments.

**Table S1.** Sequences of oligonucleotides used in this work.

| **Name**   **Sequence (5′ to 3′)** | |
| --- | --- |
| Reverse-transcriptor (H1) | TAGTAAGAATGTATAGCCCCCCCGGCTATACATTCTTACTATTTTATTT |
| Reporter (28 nt) | Chol-GCAGGGCATTCGAGCTCCT-BHQ1 |
|  | FAM-TTTTATTTA AGGAGCTCGAATGCCCTGC-Chol |
| Reporter (33 nt) | Chol-GCAGGGCATTCGAGCTCCTTTAAC-BHQ1 |
|  | FAM-TTTTATTTAGTTAAAGGAGCTCGAATGCCCTGC-Chol |
| Reporter (38 nt) | Chol-GCAGGGCATTCGACGTACGCTCCTTTAAC-BHQ1 |
|  | FAM-TTTTATTTA GTTAAAGGAGCGTACGTCGAA TGC CCTGC-Chol |
| Reporter (43 nt) | Chol-GCAGGGCATTCGACGTACTTGGCGCTCCTTTAAC-BHQ1 |
|  | FAM-TTTTATTTAGTTAAAGGAGCGCCAAGTACGTCGAATGCCCTGC-Chol |
| ssDNA (Primer) | TAGTAAGAATGTATAGCC |
| Reporter (38 nt) labeled with Cholesteryl and FAM | Chol-GCAGGGCATTCGACGTACGCTCCTTTAAC-FAM |
|  | TTTTATTTA GTTAAAGGAGCGTACGTCGA ATGCCC TGC-Chol |
| Reporter (38 nt) for LFA assays | Chol-GCAGGGCATTCGACGTACGCTCCTTTAAC-Biotin |
|  | FAM-TTTTATTTA GTTAAAGGAGCGTACGTCGAA TGCCCTGC-Chol |
| 5’-Poly-U20-crRNA | Chol-UUUUUUUUUUUUUUUUUUUUUAAUUUCUACUAAGUGUAGAUGGCUAUACAUUCUUACUA |
| 3’-Poly-U20-crRNA | UAAUUUCUACUAAGUGUAGAUGGCUAUACAUUCUUACUA UUUUUUUUUUUUUUUUUUUU-Chol |
| 5’-Poly-U5-crRNA | Chol-UUUUUUAAUUUCUACUAAGUGUAGAUGGCUAUACAUUCUUACUA |
| 5’-Poly-U10-crRNA | Chol-UUUUUUUUUUUAAUUUCUACUAAGUGUAGAUGGCUAUACAUUCUUACUA |
| crRNA | UAAUUUCUACUAAGUGUAGAUGGCUAUACAUUCUUACUA |
| HIV RNA | AAAUAAAAUAGUAAGAAUGUAUAGCC |
| SM-T | AAAUAAAAUAGUAAGAAAGUAUAGCC |
| DM-T | AAAUAAAAUACUAAGAAAGUAUAGCC |
| TM-T | AAAAAAAAUACUAAGAAAGUAUAGCC |
| HTLV-1 | CCCAAAAAACUCCAUAGGGGGGGAGG |
| HTLV-2 | GGAAAAAAACUCCUUAAGGGGGGAGA |
| HCV RNA | AGUAGUGUUGGGUCGCGAAAGGCCUUGUGGUA |
| Chol: Cholesteryl; SM-T: single-base mismatch target HIV RNA; DM-T: two-base mutant target HIV RNA; TM-T: three-base mutant target HIV RNA. Bases with underlines denote mutant bases. | |

**Table S2.** Recovery experiments of CAS-FLIER (n = 3).

| **Sample** | **Added** | **Detected** | **Recovery (%)** | **RSD (%)** |
| --- | --- | --- | --- | --- |
| 1 | 100 pM | 92.7 pM | 92.7 | 4.7 |
| 2 | 200 pM | 209.9 pM | 105 | 2.7 |
| 3 | 500 pM | 450.5 pM | 90.1 | 2.9 |
| 4 | 1 nM | 0.92 nM | 92 | 2.0 |
